# Sequence Motifs in MADS Transcription Factors Responsible for Specificity and Diversification of Protein-Protein Interaction

**DOI:** 10.1371/journal.pcbi.1001017

**Published:** 2010-11-24

**Authors:** Aalt D. J. van Dijk, Giuseppa Morabito, Martijn Fiers, Roeland C. H. J. van Ham, Gerco C. Angenent, Richard G. H. Immink

**Affiliations:** 1 Plant Research International, Bioscience, Wageningen, The Netherlands; 2 Centre for BioSystems Genomics (CBSG), Wageningen, The Netherlands; King's College London, United Kingdom

## Abstract

Protein sequences encompass tertiary structures and contain information about specific molecular interactions, which in turn determine biological functions of proteins. Knowledge about how protein sequences define interaction specificity is largely missing, in particular for paralogous protein families with high sequence similarity, such as the plant MADS domain transcription factor family. In comparison to the situation in mammalian species, this important family of transcription regulators has expanded enormously in plant species and contains over 100 members in the model plant species *Arabidopsis thaliana*. Here, we provide insight into the mechanisms that determine protein-protein interaction specificity for the Arabidopsis MADS domain transcription factor family, using an integrated computational and experimental approach. Plant MADS proteins have highly similar amino acid sequences, but their dimerization patterns vary substantially. Our computational analysis uncovered small sequence regions that explain observed differences in dimerization patterns with reasonable accuracy. Furthermore, we show the usefulness of the method for prediction of MADS domain transcription factor interaction networks in other plant species. Introduction of mutations in the predicted interaction motifs demonstrated that single amino acid mutations can have a large effect and lead to loss or gain of specific interactions. In addition, various performed bioinformatics analyses shed light on the way evolution has shaped MADS domain transcription factor interaction specificity. Identified protein-protein interaction motifs appeared to be strongly conserved among orthologs, indicating their evolutionary importance. We also provide evidence that mutations in these motifs can be a source for sub- or neo-functionalization. The analyses presented here take us a step forward in understanding protein-protein interactions and the interplay between protein sequences and network evolution.

## Author Summary

Proteins are active in complexes and therefore it is of utmost importance to understand their protein-protein interaction patterns. Over the last decade, various large-scale screening methods have been developed and implemented providing insight into interactomes for a number of model species. Despite all these efforts, our knowledge about molecular mechanisms determining interaction specificity and underlying protein network formation is limited. Here, we applied an integrated computational and experimental approach to identify short sequence motifs that determine interaction specificity for members of the large and important MADS domain transcription factor family. We show that the method is able to predict MADS domain transcription factor interaction networks for non-model plant species with a reasonable accuracy. Subsequently performed experiments revealed the possibility to rewire protein-protein interaction networks in a desired manner by generating small dedicated mutations in predicted interaction motifs. Additional bioinformatics analyses led to interesting hypotheses about protein-protein interaction network evolution. All together, these results provide insight in the way protein-protein interaction specificity is laid down in primary protein sequences and open the road towards a better understanding of protein complex formation and the possibility to control this important process.

## Introduction

Our ability to derive structural information from primary protein sequences has matured [1]–[4]. In contrast, the way in which the primary protein sequence defines protein-protein interaction specificity is still largely unknown. Nevertheless, several approaches to answer this question have recently been described [5]–[17]. These methods focus mostly on protein-peptide interactions, which are more amenable to computational and experimental analysis than interactions between full length proteins. In addition, protein structures or quantitative data about interaction energy are used, which is information that is in general not available. Though, current experimental proteomic techniques allow unraveling protein interaction networks at unprecedented scale [18]–[21] and this opens the door towards computational and experimental approaches for studying protein-protein interaction specificity at the sequence level.

Most proteins are member of protein families, which are groups of evolutionarily related proteins that are characterized by the presence of specific domains. When proteins from a given family interact with each other these interactions are often highly specific [22]. In this particular context it is even more difficult to analyze how interaction specificity is defined than in the general case, as the protein sequences show a high degree of overall similarity with various small local differences. A particular example of such a protein family is given by the plant MIKC MADS domain transcription factor family [23], [24]. The proteins from this family contain a MADS (M), Intervening (I), K-box and C-terminal domain, and have the potential to form various homo- and heterodimers [25], [26]. The MADS and K-domain are known to be involved in mediating these protein-protein interactions [27], and the I-domain is thought to be important for determination of interaction specificity [28], [29]. MADS domain protein-protein interaction specificity is tightly linked to specific functions, as exemplified by the ABC(DE) model of floral organ formation, which ascribes roles to specific combinations of MADS proteins in the development of particular floral organs [30]–[32]. The quartet model describes how floral organ identity is specified at the molecular level by the activity of four different tetrameric MADS protein complexes [33]. A second process in which specific MADS domain transcription factor complexes are involved is the timing of flowering [34], [35].

The ability to form various homo- and heterodimers is in particular relevant for eukaryotic transcription factors such as the MADS domain transcription factors [36]–[38]. Aspects of dimerization that are of functional importance include differential gene regulation by formation of dimers with distinct properties, as well as the addition of the monomer-to-dimer transition as an extra layer of regulation.

For the MADS domain transcription factor family as well as for other protein families, duplications occurred during evolution [39] which are often lineage- or species-specific. After a duplication, one of the copies experiences relaxed selection pressure and hence can evolve a specialized or new function. It is currently unclear to what extent changes in protein-protein interactions contribute to this process of sub- or neo-functionalization. Moreover, in several cases there is apparently no change in function between the two copies, leading to functional redundancy. This should be reflected in similar protein interaction patterns, even if the amino acid sequences of the proteins have diverged. Examples of redundancy in interaction patterns related to functioning have been described for members of plant MADS domain protein families [40], [41]. Similarly, examples of functional divergence related to changes in protein-protein interaction specificity have been described [42], [43]. Insight into sequence level determinants of MADS domain protein-protein interaction specificity is thus of broad biological significance.

Here we present a combined computational and experimental approach towards understanding how protein-protein interaction specificity is encoded in the MADS domain protein sequences. We started with our recently developed interaction prediction method [44], which provided a set of predicted interaction motifs. The importance of these short amino acid sequences was tested experimentally by introducing mutations and comparing the observed interaction pattern for mutated proteins with the predicted interaction pattern. We analyzed conservation and variability of those motifs, which gives insight into their role in shaping MADS domain protein functioning and the evolution of the plant MADS domain transcription factor protein-protein interaction network. At a general level, we show that protein interaction data sets can be interrogated to obtain sequence level insight into protein interaction specificity. Members of large protein families perform in general the same biochemical tasks, e.g. transcriptional regulation, but the exact biological function of each individual protein is influenced by its unique set of interactions. The approaches developed in this study set the stage for further investigations to understand how protein-protein interactions and hence protein functioning, is encoded in primary amino acid sequences.

## Results

### Predicted motifs responsible for MADS domain protein-protein interaction specificity

The basic premise of our computational approach, IMSS (Interaction Motif Search and Selection) [44], is that specific motifs in a protein sequence together determine the proteins' interaction pattern. The algorithm first searches for pairs of motifs that are overrepresented in pairs of interacting protein sequences, followed by motif pair selection via a feature selection approach. This results in a set of correlated, complementary motif pairs, that when present in pairs of protein sequences predicts whether these proteins interact. In general, a single motif does not determine the interaction specificity for a given protein, but rather this is determined by a combination of several motif pairs. A detailed overview of IMSS is given in the Methods section, including adaptations of the method made, relative to its original implementation.

We applied IMSS to all members of the *Arabidopsis* MIKC MADS domain transcription factor family, using the available interaction data [25]. Our data consists of in total 152 interactions between 35 proteins, and 478 non-interacting combinations; there is however a large spread in the number of interactions per protein, with six proteins that have only one interaction, and four that have more than 20. Previously these proteins were clustered based on their interactions [25] and although in some cases the most closely related paralogs clustered together, meaning that they have quite similar interaction specificities (e.g. AGAMOUS (AG), SHATTERPROOF1 (SHP1), SHATTERPROOF2 (SHP2) and SEEDSTICK (STK)) in other cases such paralogs did not cluster together and had quite different interaction specificities (e.g. APETALA1 (AP1), CAULIFLOWER (CAL) and FRUITFUL (FUL) or SUPPRESSOR OF OVEREXPRESSION OF CO 1 (SOC1) and AGAMOUS LIKE14 (AGL14)).

We applied IMSS with three different settings: (1) the *Arabidopsis* data as described above were used as input data with the original IMSS algorithm as presented before [44] (“ara_original”); (2) usage of “ara_original” with small modifications described in the Methods section (“ara_new”); or (3) usage of *Arabidopsis* data together with data from additional species as input (“all_species”). Leave-one-out cross-validation on the *Arabidopsis* proteins was used to select the best performing model We also tested leave-family-out cross-validation with similar results (not shown). To assess the quality of our predictions, both in the cross-validation setting discussed here and in the experimental validation described below, we use the F-score, which is the harmonic mean of precision and recall.

With 0.44+/−0.3 (average +/− standard deviation) the F-score for “ara_new” was slightly higher than the F-scores for the other two settings (“ara_original”, 0.41+/−0.3; “all_species”, 0.40+/−0.3). However, these differences are clearly very small, and indeed the overlap between the predicted interaction motifs is relatively large, especially between “ara_orig” and “ara_new”. Around 90% of the sequence positions are simultaneously either covered or not covered by a motif in each of these two models. This number is somewhat lower between these two models and the “all_species” model (around 75%). By comparing with randomly generated motif-hits with the same distribution over the proteins but randomized positions, we found that the overlaps in motifs between our three IMSS settings are statistically significant with p<0.001 (data not shown).

**Figure 1:**
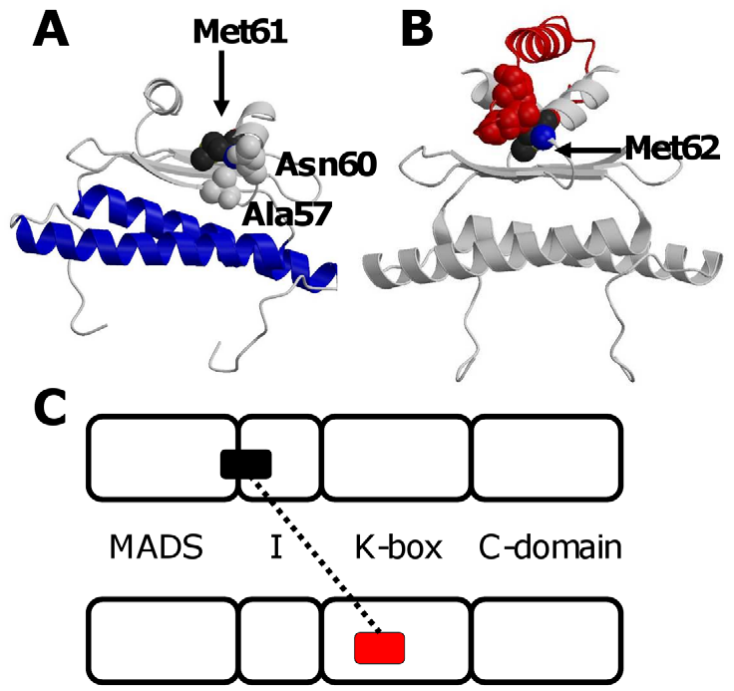
Combining predicted protein-protein interaction motifs and modeled protein structures. (**A**) Modeled dimer for the *Arabidopsis* MADS domain protein SUPPRESSOR OF OVEREXPRESSION OF CO 1 (SOC1). Blue indicates the DNA binding helix (in which no protein-protein interaction motifs are present). Residues indicated in spacefill (Ala57, Asn60 and Met61) are part of an experimentally validated interaction motif in the so-called ‘hotspot region’ (see text for details). (**B**) Crystal structure (PDB 1n6j) of human MADS domain protein MEF2 (grey) in complex with Cabin1 (red). Cabin1 contacts MEF2 via Met62 and a few other amino acid residues. MEF2 Met62 is the equivalent of Met61 in SOC1, with both amino acid residues having comparable positions in the structure. The residues of Cabin1 that contact Met62 (Ser101, Gly104 and Ile106) are shown in red spacefill. Based on the MEF2-Cabin1 structure we hypothesize a similar kind of binding of the α-helix-forming K-box from a SOC1 interacting MADS domain protein on top of the SOC1 MADS/I domain. (**C**) The black box indicates the predicted interaction motif in the ‘hotspot region’ of the SOC1 protein. The predicted complementary interaction motif (red box) is located in the K-box domain of the MADS domain protein interacting with SOC1.

For each protein the resulting predicted protein-protein interaction motifs from “ara_new” are given in Table S1. Motifs were found in all the different domains (MADS, I, K and C), but occurred most frequently at the border between the MADS and I-domain, in line with the proposed role of the I-domain in determining dimerization specificity [28], [29], [45]. This ‘hotspot’ region is homologous to a region in the human MADS domain protein Myocyte Enhancer Factor-2 (MEF2) [46] that interacts with a helix of the Cabin1 protein (Figure 1). The motif that is complementary to most of the motifs in the MIKC MADS hotspot region is found in the K-box of the interacting proteins, a domain that is predicted to form α-helices [47]–[49] comparable to Cabin1. This data suggests a specific mode of interaction between the I-region of plant MADS proteins and the K-domain of their interaction partners.

Remarkably, no interaction motifs were predicted in the first helix in the MADS domain. This helix contacts both the equivalent helix in the partner MADS domain and the DNA to which the MADS domain binds [46] (Figure 1). Hence, it is involved in protein-protein contacts, but our computational analysis predicts that these contacts do not contribute to interaction specificity. They might be important, however, in determining interaction affinity of MADS protein dimers in general. This proposed decoupling of protein-DNA and protein-protein interaction specificity determination is in line with earlier experimental observations [50] and is an example of modularity at the protein structure level, which might be important from an evolutionary perspective as it allows independent diversification of these two functions. Note however that we cannot exclude the possibility that determinants of interaction specificity are present in the first helix of the MADS domain but are missed by our approach.

In addition to the above-mentioned hotspot region, several motifs are found in the K-domain. This region is indeed known to be involved in dimerization, and in fact for a couple of proteins experimental data is available showing that mutations in the K-domain change interaction specificity. In particular, for AP3 and PI, several mutations are known that influence their partner formation [51], [52] and although IMSS does not predict motifs for AP3, for PI indeed two motifs are predicted in the K-domain (and none in the MADS or I-domain). Another example of a motif occurrence in the K-domain which can explain previous experimental observations is a motif found in CAL, which coincides with the cal-4 E131K mutation [53].

### MADS domain protein-protein interaction prediction for other species

Besides obtaining information on the molecular mechanisms underlying MADS domain protein-protein interactions, our IMSS method can be used to predict MADS domain interactomes for other plant species. In order to test the usefulness of IMSS for this purpose, interactions were predicted for MADS domain proteins of plant species from which some experimental protein-protein interaction data are available (“all_species” model; see Text S1 and Table S2). These additional data consist of 188 interactions between 98 proteins. Although, the “ara_new” model obtained the best prediction performance for *Arabidopsis* MIKC MADS domain proteins based on leave-one-out cross-validation and performed best in the experimental validation (see below), it turned out to have only a very low performance on data from non-*Arabidopsis* species, for which it obtained a recall of only 0.16. We cannot calculate precision or F-score for these additional species, because in most cases only interactions are known and no information is available about non-interactions. However, based on leave-one-out cross-validation the “all_species” model obtained a recall of 0.60+/−0.44 for the data from other species. The reason for this strong difference in performance is that despite a quite similar position of motif hits in *Arabidopsis* between the models (see above), their exact definition is somewhat different. Some of the motifs obtained upon training with *Arabidopsis* information only, appeared to be too “strict” and hence, occurring only in few cases in non-*Arabidopsis* sequences (data not shown). As mentioned above, the “all_species” model obtained a F-score of 0.40+/−0.3 for the *Arabidopsis* interactions. Hence, the performance of this model for *Arabidopsis* is only slightly lower than what was obtained without adding these additional data (“ara_new”), and the recall on these additional data is comparable to what is obtained for *Arabidopsis*. The model obtained after training with this augmented dataset provided insight into interaction patterns among family members across plant species (Tables S3 and S4) and demonstrates the usefulness of the method for the prediction of interactions of MADS domain proteins from species for which only sequence information is available.

### Validation of predicted interaction motifs

Predicted interaction motifs point to sites in the MADS protein sequence that may be essential for determination of protein-protein interaction specificity (for comparison with existing analyses see Tables S7 and S8). If this hypothesis is correct, then introducing mutations in these sites should lead to modified interaction patterns. We chose several *Arabidopsis* MIKC MADS proteins as experimental test-cases for this hypothesis. When selecting validation targets, we aimed for applying mutations to different regions in the MADS proteins and using MADS proteins with a range of different biological functions. In addition, our aim was to demonstrate gain of interaction partners, and in part we aimed for obtaining swaps of interactions partners (see Text S1). The reason for the latter is that loss of interaction partners is in principle simple to obtain by modification of the sequence, which probably affects also the folding of the particular domain in which the mutation is introduced. However, obtaining gain of interaction demands well thought amino acid changes that result in modified characteristics of an interaction site. The selected MADS domain proteins for our validation approach are involved in flowering time determination (AGAMOUS-LIKE24 (AGL24), SHORT VEGETATIVE PHASE (SVP), SOC1), floral meristem or organ identity specification (AP1, CAL and AG) or have an unknown function (AGL14). As described below, the experimental validation results for “ara_new” were somewhat better than the results for “ara_original” or “all_species”: its F-score on all mutants is 0.54 vs. 0.48 for the other two settings (we also tested whether this model was still performing best when leaving out any of the mutations, which was indeed the case for all mutations). Because of its better performance, in the presentation of the results below, we will focus on the “ara_new” results, unless otherwise indicated. Note that the slightly better performance of the experimental validation in case of “ara_new” is consistent with its better performance as judged by leave-one-out cross-validation on the *Arabidopsis* interaction data.

**Figure 2:**
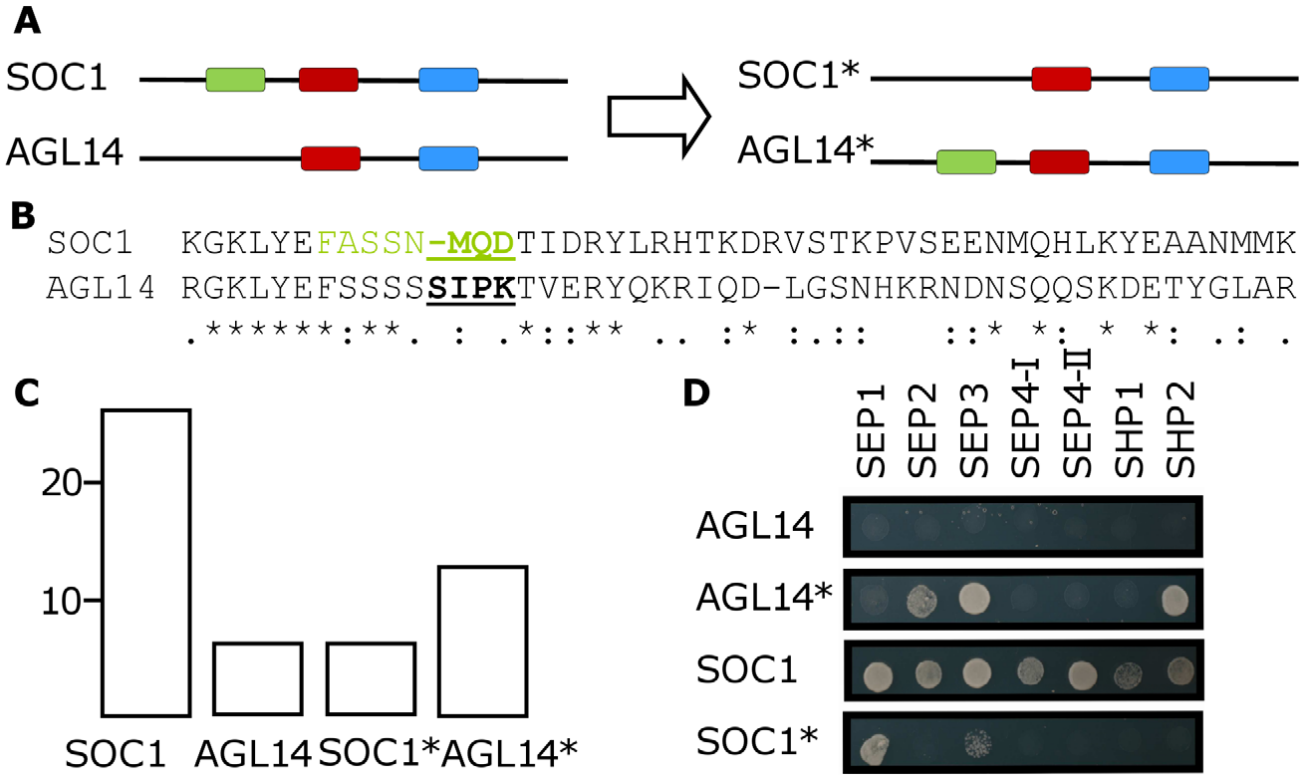
Example of the experimental validation approach. (**A**) Schematic representation of the SUPPRESSOR OF OVEREXPRESSION OF CO 1 (SOC1) and AGAMOUS LIKE14 (AGL14) MADS domain proteins. Due to the generated mutations one particular interaction motif (green rectangle) is swapped between these two *Arabidopsis* MADS domain transcription factors. The swapped motif is located in the “hotspot” region between the MADS and I-domain. (**B**) Part of a sequence alignment of AGL14 and SOC1, including the motif that was selected for mutagenesis (indicated in green in the SOC1 sequence). The mutated residues that were swapped between the two proteins are shown bold/underlined. (**C**) Bar diagrams showing the number of interaction partners for SOC1, AGL14 and their mutated counterparts SOC1* and AGL14*, respectively; see Table 1. For these two proteins, the F-scores of predicted mutant interactions are 0.63 and 0.71, respectively. (**D**) Example of a matrix-based yeast two-hybrid screen. Yeast was spotted on medium lacking Leucine, Tryptophan, and Histidine, and supplemented with 1 mM 3-amino 1,2,4-triazole to suppress transcriptional autoactivation. Growth and hence interaction events, was scored after incubation at 20°C for 4 days.

Mutations were designed based on the predicted interaction motifs (Table S1, Table S5, and Text S1) and the mutant proteins tested in a matrix-based yeast-two-hybrid assay against the collection of *Arabidopsis* MADS domain proteins [25]. The approach is illustrated in detail for the AGL14 and SOC1 proteins (Figure 2; Table 1). These two MADS proteins share over 50% sequence identity and group in the same phylogenetic clade [24], but have quite different interaction patterns. In contrast to AGL14, SOC1 contains a predicted interaction motif at the junction between the MADS domain and I-region, the position that coincides with the aforementioned ‘hotspot-region’. Swapping the amino acids in this region between SOC1 and AGL14 was sufficient to exchange a large part of their interaction specificity, as was predicted by the IMSS method. In particular, for the mutated SOC1 protein we predicted a loss of 13 of its interaction partners, and for the mutated AGL14 protein a gain of 15 (the difference of two relates to the accuracy of our method for the original interaction data). Experimentally, mutated SOC1 lost 20 of its interaction partners, and of the six that it kept, two were indeed also found as interactions partners for AGL14. Mutated AGL14 gained seven interaction partners, all of which were indeed also interacting with SOC1. As mentioned above, to assess the quality of our predictions, we use the F-score, which is the harmonic mean of precision and recall. Here, the F-score is calculated for predicting interaction patterns of mutated MADS domain proteins with the predictor that is trained with original *Arabidopsis* interaction data only. The F-score for our predictions for these two mutated proteins is 0.71 (AGL14) and 0.63 (SOC1). To put these values in perspective, we used as a null model the interaction pattern obtained by the wild type proteins. Using this null model would give a substantially lower F-score of 0.56 and 0.38, respectively, which means that our predictor performs much better in this case. F-scores for all the experimental test-cases are shown in Table S5.

**Table 1:** Interaction maps for mutated MADS domain proteins.

MADS/interactors	AG	SEP1	SEP2	SEP3	SEP4i	SEP4ii	SHP1	SHP2	AGL6	AP1	FUL	CAL	STK	XAL1	AGL13	AGL14	AGL15	AGL16	AGL17	AGL19	SOC1	AGL21	SVP1	SVP2	AGL24	FLC	MAF2	ABSI	ABSIi	AGL42	ANR1	AGL71	Loss	Gain
**SVP1**	Y	Y	Y	Y	Y	Y	Y	Y	Y	Y	Y	N	N	N	N	Y	N	Y	Y	Y	Y	Y	Y	Y	N	Y	N	N	N	Y	Y	N		
**SVP1 C58S**	**Y**	**Y**	**Y**	**Y**	**N**	*Y*	*Y*	*Y*	*N*	**Y**	**Y**	**N**	**N**	*N*	**N**	**Y**	*N*	**Y**	**Y**	*N*	**Y**	**Y**	**N**	*Y*	**N**	*Y*	**N**	**N**	**N**	**Y**	*N*	**N**	**5**	**0**
**SVP1 S61R**	**Y**	*Y*	*Y*	*Y*	**N**	**N**	**N**	**N**	**N**	*Y*	*N*	**N**	**N**	**N**	**N**	**N**	**N**	*N*	*N*	**N**	**Y**	**Y**	*N*	*N*	*N*	**N**	**N**	**N**	**N**	**N**	*N*	**N**	**15**	**0**
**SVP1 C58S/S61R**	*N*	*N*	**N**	**Y**	**N**	**N**	*N*	*N*	**N**	**N**	*N*	**N**	**N**	**N**	*N*	**N**	**N**	**Y**	*N*	*Y*	**Y**	**Y**	*N*	*N*	*N*	**N**	**N**	**N**	**N**	**N**	*N*	**N**	**17**	**0**
**SVP1 EFCSSS56-61D**	**N**	**N**	**N**	**N**	**N**	**N**	**N**	**N**	**N**	**N**	**N**	**N**	**N**	**N**	**N**	**N**	**N**	*N*	**N**	**N**	**Y**	**Y**	*N*	*N*	*N*	**N**	**N**	**N**	**N**	**N**	**N**	**N**	**20**	**0**
**SVP1 SS227-228MF**	*Y*	**Y**	*Y*	**Y**	**Y**	*Y*	*Y*	**Y**	**N**	**Y**	*Y*	*Y*	**N**	**N**	*Y*	**Y**	*N*	**Y**	**Y**	**N**	**Y**	**Y**	**N**	**Y**	**N**	*Y*	*Y*	**N**	**N**	*Y*	*Y*	**N**	**3**	**3**
**AGL24**	Y	Y	N	Y	N	N	Y	N	Y	Y	Y	N	N	N	N	Y	Y	Y	N	N	Y	Y	N	N	Y	N	N	N	N	N	N	N		
**AGL24 R61S**	**Y**	**Y**	**Y**	**Y**	*N*	**Y**	**Y**	**Y**	*N*	**Y**	**Y**	**N**	*Y*	**N**	*N*	**Y**	*N*	**Y**	**N**	**N**	**Y**	**Y**	**N**	**N**	**Y**	**N**	*Y*	**N**	**N**	*Y*	*N*	**N**	**2**	**6**
**AGL14**	N	N	N	N	N	N	N	N	N	N	Y	N	N	N	N	N	N	Y	N	N	Y	N	Y	Y	Y	N	N	N	N	N	N	N		
**AGL14 SIPK62-65MQD**	*N*	*N*	*Y*	**Y**	**N**	**N**	*N*	**Y**	**N**	*Y*	**Y**	**N**	**N**	*Y*	**Y**	**N**	**N**	**Y**	*N*	**N**	**Y**	*N*	**Y**	**Y**	**Y**	**N**	**N**	**N**	**N**	**N**	**Y**	**N**	**0**	**7**
**SOC1**	N	Y	Y	Y	Y	Y	Y	Y	Y	Y	Y	Y	N	Y	Y	Y	Y	Y	Y	Y	Y	Y	Y	Y	Y	N	N	N	N	Y	Y	Y		
**SOC1 MQD62-64SIPK**	**N**	**Y**	**N**	**Y**	**N**	**N**	**N**	**N**	**N**	**N**	**N**	*N*	**N**	*N*	**N**	**N**	**N**	*N*	**N**	**N**	**Y**	**Y**	*Y*	**N**	**Y**	**N**	**N**	**N**	**N**	*N*	**N**	*N*	**20**	**0**
**AP1**	N	Y	N	Y	N	Y	N	N	Y	N	N	N	N	N	N	N	Y	Y	N	N	Y	Y	Y	Y	Y	N	N	N	N	N	N	N		
**AP1 I66V**	**N**	**Y**	*Y*	**Y**	**N**	**N**	**N**	**N**	**N**	**N**	**N**	**N**	**N**	**N**	**N**	**N**	**N**	**Y**	**N**	**N**	**Y**	*N*	*N*	**N**	*N*	**N**	**N**	**N**	**N**	**N**	**N**	**N**	**7**	**1**
**AP1 Y148N**	**N**	**Y**	*Y*	**Y**	**N**	**N**	**N**	**N**	**N**	*Y*	*Y*	**N**	**N**	**N**	*Y*	**N**	**N**	**Y**	**N**	**N**	**Y**	*N*	**Y**	*Y*	**Y**	**N**	**N**	**N**	**N**	**N**	**N**	**N**	**4**	**4**
**AP1 I66V/Y148N**	**N**	*N*	**N**	*N*	**N**	*Y*	**N**	**N**	**N**	**N**	**N**	**N**	**N**	**N**	**N**	**N**	**N**	***N***	**N**	**N**	*N*	*N*	*N*	N	*N*	**N**	**N**	**N**	**N**	**N**	**N**	**N**	**10**	**0**
**CAL**	N	N	N	N	N	N	N	N	N	N	N	N	N	N	N	N	N	N	N	N	Y	N	N	N	N	N	N	N	N	N	N	N		
**CAL V66I**	**N**	*Y*	*Y*	*Y*	**N**	**N**	**N**	*Y*	**N**	*Y*	*Y*	**N**	**N**	*Y*	*Y*	**N**	**N**	*Y*	**N**	**N**	**Y**	**N**	*Y*	*Y*	*Y*	**N**	**N**	**N**	**N**	**N**	**N**	**N**	**0**	**12**
**CAL N150Y**	*Y*	*Y*	*Y*	*Y*	**N**	*Y*	*Y*	*Y*	**N**	*Y*	*Y*	**N**	**N**	**N**	*Y*	*Y*	**N**	*Y*	**Y**	**Y**	**Y**	**N**	*Y*	*Y*	*Y*	**N**	**N**	**N**	**N**	*Y*	**N**	**N**	**0**	**16**
**CAL V66I/N150Y**	**N**	**N**	**N**	**N**	**N**	**N**	**N**	**N**	**N**	**N**	**N**	**N**	**N**	**N**	**N**	**N**	**N**	**N**	**N**	**N**	*N*	**N**	**N**	**N**	**N**	**N**	**N**	**N**	**N**	**N**	**N**	**N**	**1**	**0**
**AG**	N	Y	Y	Y	N	N	N	N	N	N	Y	N	N	N	Y	N	Y	Y	N	N	N	Y	Y	Y	Y	N	N	N	N	N	N	N		
**AG Q126H**	**N**	**Y**	*Y*	**Y**	**N**	**N**	**N**	**N**	**N**	**N**	**N**	**N**	**N**	**N**	**N**	**N**	**N**	**Y**	**N**	**N**	*N*	*N*	*N*	**N**	*N*	**N**	**N**	**N**	**N**	**N**	**N**	**N**	**7**	**0**

Results from the comprehensive matrix-based yeast two-hybrid screening of all mutated proteins against the complete collection of *Arabidopsis* MIKC MADS domain proteins [25]. Note that only MIKC proteins are shown that interact with at least one of the mutated proteins. Non-interacting combinations are listed only for the MIKC proteins in the table. All possible not listed combinations are non-interacting. The screening was done in duplicate. For each generated mutant protein also the interaction data from the wild type protein from which it originates is indicated for comparison. In the last two columns loss and gain of interactions in comparison to the non-mutated proteins is indicated. Y  =  interacting in yeast-two hybrid assay; N  =  not interacting in yeast-two hybrid assay. Correct predictions are indicated in bold and incorrect predictions in italics.

**Figure 3:**
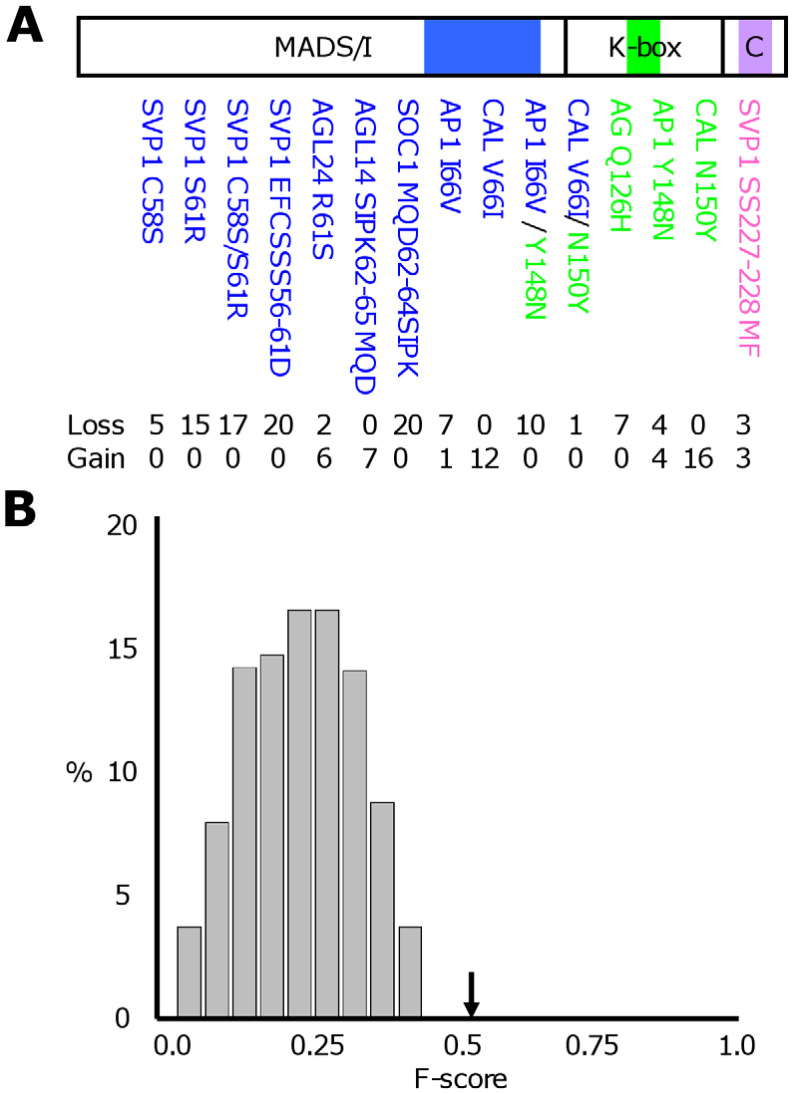
Effect of motif-based mutations on interaction patterns. (A) Mutations were introduced based on predicted interaction motifs as explained in Figure 2. Different domains in MIKC MADS domain proteins are shown with colored boxes indicating the various regions in which point mutations were introduced. Below these, the various mutant MADS domain proteins that were generated are listed. The descriptions of the mutated proteins are colored based on the domain in which the mutation was generated. The mutated MADS domain proteins are SHORT VEGETATIVE PHASE (SVP1), AGAMOUS LIKE 24 (AGL24), SUPPRESSOR OF OVEREXPRESSION OF CO 1 (SOC1), APETALLA1 (AP1), CAULIFLOWER (CAL), and AGAMOUS (AG). Note that there are two double mutations for which one mutation occurs in the MADS/I domain and one in the K-box. Below each mutated protein, the number of losses and gains of protein-protein interactions in the yeast two-hybrid assay for the mutated proteins in comparison to the native MADS domain proteins is indicated (see Table 1 for interaction partner identities). (**B**) Histogram of F-scores for prediction of effect of mutants based on randomized input data (see text for details). The arrow indicates the F-score obtained by the predictor trained on experimental input data.

All mutations that we introduced led to changes in interactions. Overall, in eight out of 15 cases (∼53%) the mutations we introduced led specifically to loss of interactions, in three cases (∼20%) specifically to gain of interactions, and in the remaining ∼27% of cases, both loss and gain were obtained (Figure 3, Table 1, and Table S6). The number of gains and losses that our IMSS method (“ara_new” model) predicted, displayed a good correlation with the experimentally observed number of gains and losses (Pearson correlation coefficient 0.76, p-value 0.0005). When separating gains and losses, the correlation coefficient values are 0.63 and 0.67, respectively (p-value<0.006).

The changing interaction patterns were predicted with a reasonable accuracy for most of the mutated proteins (Figure 3B; Table 1). In particular, only for five out of 15 cases our predictions had a worse F-score than the F-score obtained using the original interaction pattern as predictor for the mutated proteins (null model). For seven out of 15 mutations our predictions had a better F-score and for an additional three cases, the obtained F-scores were similar to each other. These latter cases all concerned mutations in the CAL protein, which were introduced based on results of the “ara_original” model, but that in the “ara_new” model were predicted to have no effect. Surprisingly, the effect of the CAL double mutation was that no interaction remained at all. Almost the same holds for the AP1 double mutation (only one interaction remained). One particular explanation for these results could be that introduction of double mutations has the unfortunate side-effect of disturbing the structure of the proteins to a large extent. This might in particular be the case when these two mutations, which are far apart in the sequence of those proteins, coincide in the 3D structure of the protein. Although no structure data is available for these proteins outside the MADS domain, we investigated this possibility by performing an intramolecular correlated mutation analysis on a large set of AP1 orthologs (for CAL, too few orthologous sequences were available). The analysis predicted several intramolecular interactions of residues in the K-box helix around the mutation side with residues in the I-region around the mutation side, e.g. residue E149 in the K-box with residue S74. Based on this observation, we hypothesize that double mutations in AP1 and CAL (which is closely related to AP1) introduce too large structural changes leading to instability or mis-folding of the protein. Hence, the fact that our predictions are not correct in this case can be rationalized.

In SVP we introduced two single mutations and a double mutation aiming to change its interaction pattern towards that of the closely related AGL24 proteins. Alternative splicing has been reported for SVP and the protein named SVP1, which was used in this particular experiment, is encoded by the fully spliced SVP transcript [54]. Although SVP and AGL24 are phylogenetically grouped in the same clade [24], they act opposite in flowering, being a repressor and activator of this process, respectively. In this case the two mutations are in close proximity in the sequence and within one predicted interaction motif positioned at the ‘hotspot region’ between the MADS and I domain of the SVP protein. Swapping of this interaction motif between AGL24 and SVP1 did not result in a complete exchange of interaction pattern (Table 1). Though, for one single mutation (SVP1 S61R) and the double SVP1 mutant (SVP1 C58S/S61R) at least interaction with the floral repressor protein FLOWERING LOCUS C (FLC) was lost, which is supposed to be important for its floral repressor function [55]. Introducing the “SVP1”-motif in AGL24 did not result in interaction with FLC, but the mutated protein (AGL24 R61S) gained interaction with MADS AFFECTING FLOWERING 2 (MAF2), which is closely related to FLC and acts also as floral repressor [56]. Probably, mutations in various interaction motifs need to be combined in order to get an exact and complete swap of interaction pattern between AGL24 and SVP1. Indeed, mutations in predicted interaction motifs in the C-terminal region of SVP1, which is most divergent from AGL24, showed that this part of the protein also contributes to SVP1 interaction specificity (Table 1).

As indicated above, our predictor performs worse than the null model in five out of 15 cases. This seemingly moderate performance can be understood as caused by side effects of the mutations for at least some of the cases (see discussion about AP1 and CAL above). Nevertheless, in order to further investigate the statistical significance of our predictions of interaction changes upon introducing mutations and to judge the value of our predictor, we used a second null model. Here, a series of models were trained with interaction data to which increasing amounts of noise were added, from 10% to 40% (see Methods for details). If our predictions using the experimental data are significantly different from random expectation, one would expect that the more noise is added to the data, the worse the prediction is. This is indeed what we observe, with the F-score dropping continuously from 0.46+/−0.05 at 10% noise to 0.24+/−0.1 at 40% noise (average and standard deviation over 10 different random tests). As an extreme version of this randomization we also performed the test using fully randomized data as input, obtained by keeping the interaction network unchanged but randomly reassigning sequences to the MADS proteins. This randomization was repeated 1000 times, resulting in an average F-score of 0.23+/−0.1. None of these random tests obtained a similar or higher F-score than what was obtained for our predictor trained with the experimental data (Figure 3B). This indicates that with p<0.001 our results are significantly different from random expectation.

### Evolutionary dynamics of interaction motifs

The putative effect of mutations on protein-protein interaction specificity must have played an important role in the evolution of the MADS protein interaction network. Our interaction motifs provide means to investigate this. Of importance here is the balance between motif conservation, which leads to a static interaction network, and variability of motifs, which could lead to interaction loss or gain and potentially to neo- or sub-functionalization of particular proteins. The latter is especially relevant after gene or (partial) genome duplications, which has played an important role in expansion of the plant MADS domain transcription factor family [39]. We expect to observe overall conservation of the interaction motifs, but also variation of motifs when comparing duplicated proteins. We performed several analyses on the interaction motifs to test this hypothesis, the results of which are described below.

### Motif conservation

**Figure 4:**
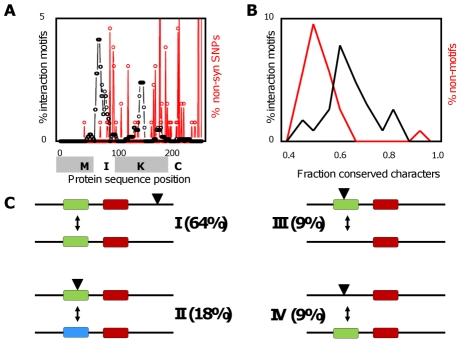
Interaction motifs and network evolution. The role of conservation of interaction motifs versus variation of these motifs was investigated. (**A**) Histogram of occurrences of interaction motifs (black) and SNPs (red) at particular positions in the protein sequences of all *Arabidopsis* MIKC MADS proteins. Note that there is hardly any overlap between interaction motifs and SNPs. Positions of the M, I, K and C domain are indicated. (**B**) Histogram of cross-species conservation of interaction motifs (black) and non-motif-sequences (red) in MIKC MADS domain protein sequences. Non-motif sequences are defined at positions in MADS protein sequences where in other MADS sequences a motif is present. (**C**) Four different scenarios are possible if after duplication of a MADS domain protein sequence an indel occurs in one of the two sequences: (I) indel does not overlap with a predicted interaction motif; (II) both insertion and deletion overlap with a motif; (III) only insertion or (IV) only deletion overlap with a motif. Lines indicate sequences, colored boxes indicate predicted interaction motifs, triangles indicate insertion, and arrows indicate effect of insertion/deletion on motif. As discussed in the text, if an indel overlaps a motif (scenario II-IV), in half of the cases (18% for scenario II vs 9% for each of scenario III and IV) it does not delete but only modifies the motif (illustrated by a change in color for the motif).

First, we compared predicted motifs with available non-synonymous single nucleotide polymorphism (SNP) data [57]. Comparison of “motif density” and “SNP density” showed that these are negatively correlated (Figure 4A). For the ∼1500 non-synonymous SNPs falling within MADS protein sequences, 170 cases were found where a SNP was located inside a motif occurrence (Table S10). Randomly generated motif occurrences with the same number of occurrences per protein as the predicted motifs were generated in 1000 trials. The average overlap of SNPs with those motif occurrences was 351+/−116, and in 965 out of 1000 random trials the overlap was larger than 170. This indicates that the experimental overlap between IMSS motifs and SNPs is significantly smaller than the randomly expected overlap (p< = 0.04; see Table S10). In addition, the cases where SNPs overlap motifs are conservative mutations (several non-conservative SNPs do affect the MADS proteins, but they fall outside the predicted interaction motifs). In fact, the largest contribution to the 170 overlaps between SNPs and motifs is formed by in total 122 SNPs found at two consecutive positions in AGL14, where an S is changed to a T and a T to an S (Ser187, Thr188). The few cases with an overlap between a potentially more important SNP and an interaction motif indicate interesting candidates for putative causes of functional differences between MADS proteins in various *Arabidopsis* accessions. This includes for instance a Q->E SNP in ANR1 that occurs in several ecotypes (Table S10).

The analysis above only captures short term evolutionary dynamics and might be biased by the preference of motif existence in certain sequence regions (e.g. the motifs are generally absent in the C-region of the proteins, which is the most variable region). To obtain further insight into the conservation of predicted interaction motifs, we analyzed the extent to which motifs are conserved in putative orthologous protein sequences from various sequenced plant genomes (see Methods). This conservation was compared to that of homologous regions in MADS proteins without a motif occurrence at that particular position in the sequence. This approach ensures that the results are not influenced by a bias for motifs in particular protein domains. The analysis showed that on average the fraction of motif characters that were completely conserved was 0.65+/−0.09, whereas the same fraction for non-motifs was 0.55+/−0.09 (average +/− standard deviation). We used a Kolmogorov-Smirnov test which indicated that with p∼10^−7^ the distribution of fraction conservation of characters of motifs was significantly different from that of non-motif characters. Focusing on motif-characters that are non-wildcards (i.e. not “*”), the difference was even somewhat higher, 0.73+/−0.09 (motifs) vs. 0.58+/−0.07 (non-motifs) (Figure 4B). In addition, the number of non-conservative changes was much higher in the non-motifs than in the motifs; for example, a swap from K or R to D or E occurred in only 1% of the characters of motifs versus 3% of the non-motif sequence regions. Note that our interaction motifs were obtained using a limited set of sequences and interaction data, and as such their conservation across various species is an independent validation of their functional importance.

### Motif variability

The observed conservation of predicted interaction motifs provides supporting evidence that the IMSS motifs are under functional constraints. Such functional constraints would be relaxed after a gene duplication in one of the two copies, allowing sub- or neo-functionalization to occur via modification of interaction motifs. To analyze this, we first focused on indels coinciding with IMSS motifs (see Text S1; note that here we used motifs from the “all_species” model as these are more appropriate in this context where we use sequences from various species). This revealed several examples of duplicated proteins where an indel overlaps with an interaction motif (Table S11). Specifically, out of 81 pairs of putative paralogous proteins containing an indel, 29 pairs were found in which the indel overlaps with a predicted interaction motif (in 20 different species). In half of these cases both of the paralogs contain a motif (albeit in general a different one) at the indel position (Figure 4C). Those cases where both copies contain an interaction motif are the result of sequence changes after the gene duplication that modified, but did not delete the interaction motif. This suggests that after such duplication one of the copies indeed acquired a novel or specialized function (option II in Figure 4C). Hence, this analysis highlights interesting candidates for possible sub- or neo-functionalization and confirms the expected role of variability of interaction motifs in these processes. Unfortunately, in most cases we do not know the interaction patterns of the proteins so we cannot validate our predictions, but the *Arabidopsis* cases we found have clearly different interaction patterns. For the proteins in the SEP clade for instance, some evidence is present that they are not fully functional redundant [58], [59].

### Neo- and sub-functionalization

**Figure 5:**
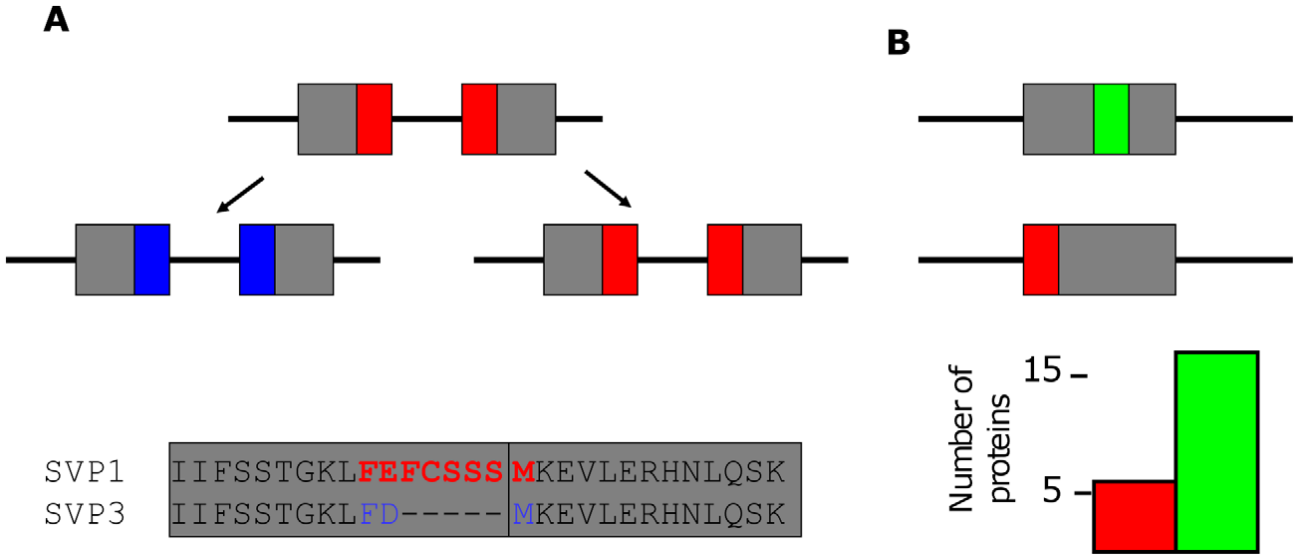
Mechanism of generating protein-protein interaction diversity by shifting intron/exon borders. (**A**) After a duplication of a gene or in the case of alternative splicing, a shift of an intron/exon border can modify a protein interaction motif which overlaps or is close to such a border. *Top panel,* schematic illustration of this process. Line indicates gene sequence, grey bars indicate exons, and colored bars indicate predicted interaction motifs. *Bottom panel*, part of a protein sequence alignment for the *Arabidopsis* MADS domain protein SHORT VEGETATIVE PHASE (SVP1) and an identified alternatively spliced SVP form named SVP3. A predicted interaction motif in SVP1 which is almost completely spliced out in SVP3 is shown in red. Two grey bars indicate the two adjacent exons. (**B**) Predicted interaction motifs can be either close to an intron/exon border (indicated by red motif) or far away from the intron/exon border (green motif). Bars in the graph indicate average number of *Arabidopsis* MIKC MADS proteins in which predicted interaction motifs occur for two different motif groups: motifs that are located close to the intron/exon border (<3 amino acids distance, red) occur on average in a few proteins only, and motifs that are located far away from the border (> = 3 amino acids distance, green) occur in many proteins.

Interestingly, the interaction motifs in the ‘hotspot region’ at the junction between MADS and I domain overlap an intron/exon boundary. This provides a plausible evolutionary mechanism to generate protein interaction diversity by shifting these intron/exon boundaries after duplications or via alternative splicing (Figure 5). Indeed the above mentioned cases where indels modify interaction motifs in duplicated proteins occur often in the MADS domain protein ‘hotspot region’, in which interaction motifs overlap an intron/exon boundary. An example of a change in interaction pattern via ‘splicing out’ of a predicted interaction motif is observed in a recently identified *SVP* splicing variant (named SVP3; Accession: EU078686; Figure 5). SVP3 lost the interaction motif found in the hotspot region of the fully spliced SVP protein (SVP1) leading to a large loss of protein interaction partners (Figure 5; Table 1; Figure S2). Additional discussion is provided in the Supplementary information (Text S1, Figure S1), where we also provide an analysis of the distance of predicted interaction motifs from intron/exon boundaries. Based on these findings we hypothesize that shifting intron/exon borders plays a role in neo-functionalization of plant MADS domain transcription factors by direct changing of dimerization capacity (Figure 5). Note that, at least in the above-mentioned SVP case, this mechanism seems to allow the duplicate to optimize in one specific interaction and avoid conflict with the original copy, by deleting other common interactions.

A clear example of the effect of sub- or neo-functionalization for MADS proteins is given by the LpMADS1 protein from *Lolium perenne*, a grass species. Based on overall sequence similarity, this protein is part of the *Arabidopsis* AP1 clade. However, the interactions and expression pattern of LpMADS1 clearly resemble *Arabidopsis* SOC1 [60], and our interaction motif prediction supports this: it correctly predicted most (14 out of 16) of the interaction partners of LpMADS1, including several which are not interacting with AP1 (e.g. SEP2, AGL14). In our predictor (using the “all_species” model) LpMADS1 contains two specific motifs which SOC1 also contains, but AP1 does not (in addition LpMADS1 contains a set of motifs which it shares with both AP1 and SOC1). One of these is located in the C-terminus region, and overlaps with a motif which we experimentally targeted in SVP1 (SS227-228MF mutation in SVP1). Indeed, the experimental validation showed that mutations in this region influence interaction specificity (Table 1).

As a further example, sequences for various *Arabidopsis* SEP homologs were analyzed and the occurrence of a specific interaction motif in the above mentioned ‘hotspot’ region was found to correlate with differences in expression for the genes encoding those proteins (Text S1, Table S9). This again supports the proposed importance of variations in interaction motifs as a means to sub- or neo-functionalization.

## Discussion

Our method predicting sites mediating protein interaction specificity, and our experimental data showing changes in interaction specificity extend previous examples, in which protein-protein interactions were modified through mutation of a few amino acids or even a single amino acid [5], [6], [61], [62]. In particular, we now demonstrated this for a family of interacting proteins, for which interaction specificity is governed by subtle differences in their sequences. Moreover, we performed various additional computational analyses for the predicted interaction motifs which support their importance.

Our IMSS approach is not perfect, but it is one of the first to approach computationally the important problem of interaction specificity in a paralogous family of interacting proteins. The method is based on the occurrence of combinations of motifs and offers a number of advantages over currently available tools (which are not specifically targeted towards the context of interaction specificity in a paralogous family of interacting proteins). In contrast to methods such as InSite [63], IMSS does not rely on a pre-selection of conserved sequence motifs from a database, and in contrast to correlated mutations approaches [6], [64]–[66], our method does not need accurate alignments. In cases where a quantitative model of interaction energetics is available, a method such as CLASSY [5] might be applicable, but there are many protein families such as the MIKC MADS proteins, for which such data is not available. Also, our approach does not need protein structure data, which is important as protein structure information is lacking for many protein families. Nevertheless, our method also has its own weaknesses, one of which is that we have to rely on existing interaction data which we use as training data, but it is clearly complementary to existing approaches. In this study we focused on analysis of the properties of our predicted interaction motifs, but we also demonstrated the usefulness of our method for the prediction of protein-protein interactions for MADS domain proteins from other plant species, including crops. For most of these only sequence information is available and knowledge about interaction capacity of MADS domain proteins is completely lacking. Here, our method provides an alternative and orthogonal way to predict interactions, as compared e.g. to the ‘interolog’ approach [67], [68].

Traditional experimental techniques to identify and test the role of specific amino acids or combinations of amino acid residues in specifying protein-protein interaction capacity, such as “alanine scanning” (e.g. [69]), are based on systematic mutational analyses and therefore, laborious. In contrast, the combined computational and experimental approach we followed is a fast way to pin-point motifs putatively involved in determining interaction specificity. We demonstrated that the predicted motifs can be targeted to change MIKC MADS protein interaction specificity and hence rewire the interaction network. A problem we encountered is that mutations can have unwarranted side effects on e.g. the structure of the proteins involved. Probably, this can be solved in the near future by a combination of IMSS with detailed protein structure modeling. Furthermore, in some cases mutations lead to novel interaction patterns that do not occur for any MADS domain protein in the original family. Remarkably, this is at least partially predicted correctly by our IMSS method. The result is complementary to the recent observation that bZIP-like coiled-coil proteins only sparsely sample the possible interaction space [5] and suggests that it is a common scenario for protein-protein interaction networks.

Studies of gene regulatory network evolution often focus on *cis*-regulatory changes [70], which may affect only part of the activity of a factor and often lead to sub-functionalization after a duplication event. Changes in coding regions have a higher chance of leading to non-functionalization. However, they may also generate completely new functions due to changes in e.g. interaction patterns, which in turn may lead to sub- or neo-functionalization for one of the two copies after a duplication. We have shown that the predicted interaction motifs are important for the protein interaction specificity of the MIKC MADS domain proteins and that even single amino acid mutations in these motifs result in either a gain or loss of interactions in a predictable way. This data suggests that these motifs underlie neo-functionalization in the MIKC MADS family, which is a transcription factor family that has been heavily expanded in plants during evolution through both whole genome- and small-scale duplications [39]. The duplicated genes were recruited for novel developmental networks, e.g for regulating the formation of the floral organs, although they were also subjected to sub-functionalization and many are therefore still acting in a (partially) redundant manner [41], [71]–[73]. Our results represent a step forward in understanding how proteins perform their molecular function. The challenge ahead for the MADS domain transcription factor family and transcription factors in general is to extend this method to higher-order protein complexes [33], [74]–[76], interactions with non-MADS interaction partners, and protein-DNA interactions [77], [78].

## Methods

### Interaction Motif Search and Selection (IMSS)

The basic idea behind our bioinformatics method to predict protein interactions based on their sequences was recently published [44]. Here we give a brief overview and present modifications to the algorithm. As a first step in our approach we used the correlated motif search algorithm D-STAR [79]. This finds correlated motifs that are over-represented in pairs of interacting protein sequences. Correlated motif pairs are defined over pairs of sequences; this means that for each protein-protein interaction pair we can define absence or presence of particular correlated motif pairs. Next, we used these motifs together with experimental interaction data to train a Random Forest [80] classification algorithm in combination with a feature-selection procedure [81]. In the latter, previously the accuracy of prediction was used as scoring criterion, but in order to deal better with unbalanced datasets we now changed this to the F-score. In addition, before running the feature-selection, we now first performed a simple clustering step of motifs using the algorithm described in [82]. This was based on the similarity of occurrences of motif pairs (number of sequence pairs in which both motifs occur divided by number of sequence pairs in which at least one of the two motifs occurs). These modifications were not used in the “ara_original” model but were applied in the “ara_new” and the “all_species” model.

### Experimental validation

IMSS-prediction identifies regions in the protein, but does not directly tell which amino acid residue to change in the short motif sequence, and into which amino acid it has to be changed. In order to decide on this, we aligned sequences of related proteins and chose residues to be mutated based on conservation (See Text S1). The residues selected for amino acid mutation are listed in Table S2.

The point mutations were generated by PCR-based site-directed mutagenesis using the original Gateway entry clones [24] containing the respective MADS domain ORFs as a template. For each gene a forward primer was generated at the start codon and a reverse primer at the stop codon. In addition, two complementary primers were made that anneal to the region where the mutation has to be introduced and which contain the altered nucleotides encoding the mutated amino acids. Initially, two PCR reactions were performed with proofreading Phusion DNA polymerase (Finnzymes, Finland). The forward primer at the ATG and the reverse primer at the position of the mutation were used in the first PCR, while the forward primer at the position of the mutation and the reverse primer at the stop codon were used in the second PCR. The two purified fragments were used as template in a PCR reaction with the forward and reverse primer on the start and stop codon, respectively. The complete ORFs were cloned into the pCR8/GW/TOPO Gateway entry vector (Invitrogen, Carlsbad, US). The presence of the mutation was confirmed by sequence analysis (DETT sequencing kit Amersham, GE Healthcare, UK). Finally, the mutated ORFs were cloned into the pADGAL4 (pDEST22) and pBDGAL4 (pDEST32) Gateway destination vectors (Invitrogen, Carlsbad, US) by LR reactions.

Yeast transformation was performed according to the Quick and Easy transformation protocol [83]. Although, from the proteins selected for mutation only a low level of transcriptional activation activity was shown previously for the AP1 protein [25], all generated mutated proteins expressed as GAL4-BD fusion proteins were first tested for auto-activation capacity. For this purpose, the yeast clones were spotted onto selective SD medium lacking Leucine and Histidine and supplemented with a range of 3 Amino1,2,4-Triazole (3AT; 1, 5, and 10 mM). Growth of yeast and hence autoactivation, was scored after 5 days incubation at 22 degrees Celsius. These analyses revealed that all mutated proteins behave like the wild type protein from which they originate and none contains a strong transcriptional activation domain. In theory proteins fused to the GAL4 AD domain can also give activation of yeast reporters, when the protein is able to bind to regulatory sequences of the reporter genes. We didn't screen for this possibility, but the fact that none of the generated AD-GAL4 fusion proteins was giving growth of yeast in the final screening for all or almost all tested combinations, shows that none of them is auto-activating as GAL4-AD fusion protein. Subsequently, matrix-based two-hybrid screenings were performed in duplo and by scoring for at least two different reporter genes, as previously described [25]. Combinations were scored as interactions when giving growth for one of the selection markers in both screens and both selection markers in at least one of the screens.

### Statistical analyses

Performance of our predictions of changes in interaction specificity was measured via the F-score, which equals 2*precision*recall/(precision+recall). Here precision  =  TP/(TP+FP) and recall  =  TP/(TP+FN), where TP indicates the number of correctly predicted interactions, FP indicates the number of predicted interactions that are not correct, and FN indicates the number of experimental interactions that are incorrectly not predicted as interactions.

The F-score when using the original interaction data as a predictor for the interactions of the mutated proteins was calculated in a similar manner. In addition, a series of models were trained with interaction data to which increasing amounts of noise were added, from 10% to 40%. These numbers refer to the percentages of interactions that were removed from the data, and converted into interactions between randomly chosen pairs of proteins that do not interact according to the experimental data.

### Structural and evolutionary analysis

Protein structures were modeled using Modeller 8.2 [84], with the structures 1EGW [85] and 1N6J [46] as templates, using the automodel module and generating 1000 structures. The best one according to objective function was selected. SNP data were obtained from www.1001genomes.org (data for 80 ecotypes). Intron/exon structures were defined using the software tool Scipio [86]. To compare the observed overlap of predicted motifs with SNPs or their observed distance from intron/exon boundaries with random expectation, random motif instances were generated by randomly choosing a number of motif locations in each protein equal to the predicted number of motif locations. This was repeated 1000 times for each sequence.

Conservation of motif occurrences was assessed as follows. First, MIKC MADS sequences were obtained from the genomes of rice [87], poplar [88], grape vine [89], maize (www.maizesequence.org), *Medicago truncatula* (www.medicago.org), papaya [90] and sorghum [91]. For rice and poplar we used the MIKC MADS domain protein sequences as provided in the respective publications; for the other genomes, sequences were obtained from the full set of coding sequences using the profile HMM software HMMER [92] with the PFAM [93] models for the MADS-domain (SRF-TF) and the K-domain (K-box). Next, putative orthologs for the *Arabidopsis* protein sequences were identified by aligning each sequence to each *Arabidopsis* sequence using MUSCLE [94] and using sequence identity as the criterion in a bi-directional best hit approach. Subsequently, for each motif occurrence in a particular *Arabidopsis* protein, its conservation was calculated as the fraction of characters in the motif which were identical in the homologous regions in its orthologs; the same was calculated for all *Arabidopsis* proteins which did not have a motif occurrence at that particular location in the sequence.

To obtain insight into the dynamics of interaction motifs upon duplications, we analyzed a set of 1,459 MIKC MADS domain protein sequences from 257 species (obtained from Interpro by requiring the presence of both a MADS and K-box domain, IPR002487 and IPR002100, respectively). From these, we obtained pairs of putative duplicates, which we defined simply as two proteins from the same species both having their highest sequence similarity with members of the same clade in *Arabidopsis* (as defined in [95]).

We focused on indels because occurrence of an insertion or deletion could be interpreted as a signature of disruption of the interaction motif. For each pair of protein sequences, indel positions in their sequence alignments were probed by looking for stretches of length *d* with high sequence identity, and one insertion/deletion occurring. *d* was set to 6, and the cutoff for identity was set to 5 (i.e. all positions except the indel were required to be identical). Subsequently, the overlap between those indels and the predicted interaction motifs was assessed.

To perform intramolecular correlated mutation analysis of AP1, sequences of MADS proteins were obtained using blastp on the NR database, filtering with hmmsearch [92] to retain only sequences with a MADS-domain and a K-domain, and assigning sequences as putative AP1 orthologs using a best-hit criterion. These sequences were aligned with MUSCLE [94]. Subsequently, the CAPS [96] algorithm was used to obtain correlated mutations, using a reasonably stringent cutoff of 0.4 on the Pearson correlation coefficient that is returned between pairs of sites with this algorithm.

## Supporting Information

Figure S1 Relation between position of intron-exon borders and predicted interaction motifs. Histogram of distances (amino acids, X-axis) between motif occurrences and exon borders, for IMSS motifs (red) and random motif occurrences (green).
(9.45 MB TIF)

Figure S2 Effects of alternative splicing of SHORT VEGETATIVE PHASE (SVP) and SEPALLATA4 (SEP4) on predicted interaction motifs. Top, in alternatively spliced SVP3 (SHORT VEGETATIVE PHASE, splicing variant three), a predicted motif at an exon border is spliced out, resulting in loss of interactions (bold indicates motif occurrence in SVP1). Bottom, splicing removes predicted interaction motifs from the SEP4-II splice variant (SEPALLATA4-II) that are present in the SEP4-I variant (bold indicates motif occurrences in SEP4-I).
(0.31 MB TIF)

Table S1 IMSS motif positions within the Arabidopsis MIKC MADS domain transcription factor proteins.
(0.55 MB DOC)

Table S2 Interaction data from additional species.
(0.20 MB DOC)

Table S3 Prediction of large-scale MADS interaction data.
(0.04 MB DOC)

Table S4 Interaction prediction for SEP-homologs.
(0.05 MB DOC)

Table S5 Mutagenesis positions.
(0.06 MB DOC)

Table S6 Results yeast two-hybrid assays.
(0.48 MB DOC)

Table S7 Analysis of known mutations in MADS domain proteins.
(0.08 MB DOC)

Table S8 Previous computational studies that revealed residues that are under functional constraint.
(0.05 MB DOC)

Table S9 SEP equivalents grouped according to expression in whorl one.
(0.05 MB DOC)

Table S10 SNPs overlapping IMSS motifs.
(0.05 MB DOC)

Table S11 Pairs of sequences with indel overlapping predicted interaction motif.
(0.07 MB DOC)

Text S1 Additional text van Dijk et al.
(0.11 MB DOC)
