# Supplementary figures and images for: Sequence Motifs in MADS Transcription Factors Responsible for Specificity and Diversification of Protein-Protein Interaction
Source: PLoS Comput Biol. 2010 Nov 24;6(11):e1001017. doi: 10.1371/journal.pcbi.1001017 (PMC2991254; doi:10.1371/journal.pcbi.1001017)

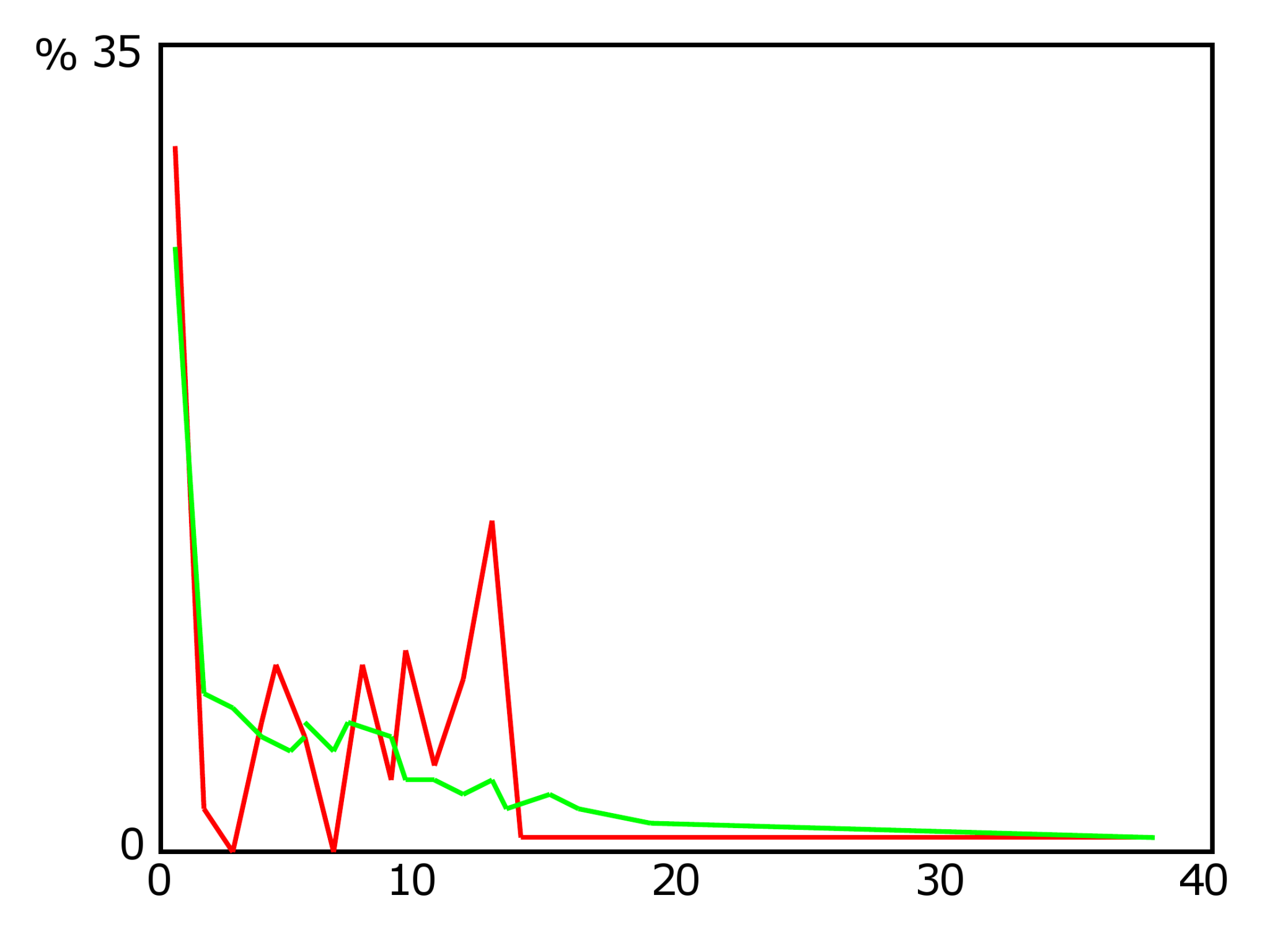

Supplement: Figure S1 — Relation between position of intron-exon borders and predicted interaction motifs. Histogram of distances (amino acids, X-axis) between motif occurrences and exon borders, for IMSS motifs (red) and random motif occurrences (green). (9.45 MB TIF) [file pcbi.1001017.s001.tif]

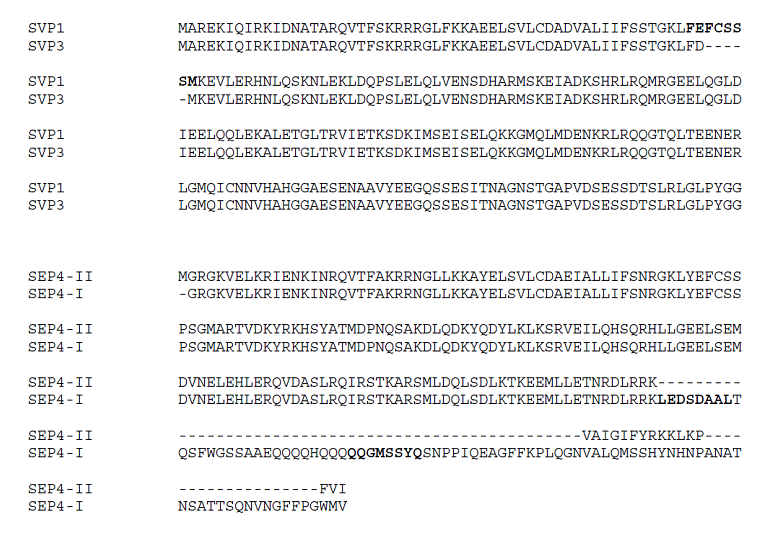

Supplement: Figure S2 — Effects of alternative splicing of SHORT VEGETATIVE PHASE (SVP) and SEPALLATA4 (SEP4) on predicted interaction motifs. Top, in alternatively spliced SVP3 (SHORT VEGETATIVE PHASE, splicing variant three), a predicted motif at an exon border is spliced out, resulting in loss of interactions (bold indicates motif occurrence in SVP1). Bottom, splicing removes predicted interaction motifs from the SEP4-II splice variant (SEPALLATA4-II) that are present in the SEP4-I variant (bold indicates motif occurrences in SEP4-I). (0.31 MB TIF) [file pcbi.1001017.s002.tif]
