# Supplementary material for: Sequence Motifs in MADS Transcription Factors Responsible for Specificity and Diversification of Protein-Protein Interaction
Source: PLoS Comput Biol. 2010 Nov 24;6(11):e1001017. doi: 10.1371/journal.pcbi.1001017 (PMC2991254; doi:10.1371/journal.pcbi.1001017)
Supplement: Table S1 — IMSS motif positions within the Arabidopsis MIKC MADS domain transcription factor proteins. (0.55 MB DOC) [file pcbi.1001017.s003.doc]

**Table S1. IMSS motifs and motif positions within the *Arabidopsis* MIKC MADS domain transcription factor proteins**

**S1.A. IMSS motifs**

| Motif number^a^ | Motif ^b^ | Domain^c^ |
| --- | --- | --- |
| 1A | [IV]FSXXX[KR]L | I |
| 1B | [ED]FXSSXMX | I |
| 2A | PSSXVXTX | C |
| 2B | XFSXXGXL | I |
| 3A | [EKIL][EA][LQ]XX[LIV][EL]X | C |
| 3B | MXXX[IL][DE]RY | K |
| 4A | XXG[KR]L[FY][ED]X | I |
| 4B | FXSSSX[KM]X | K |
| 5A | F[AS]SSXMXX | K |
| 5B | [LV]X[IVE][FY]SXX[GD] | I |
| 6A | [SN]SSX[KS]XX[IL] | K |
| 6B | [LV]X[IV]FSXX[GS] | I |
| 7A | XELQXXXX | C |
| 7B | FXSSSX[KM]X | K |
| 8A | SXKXXXXR | K |
| 8B | [EK][EA]LXX[LI][ED]X | C |
| 9A | L[QE]XXXXXL | C |
| 9B | XXXSSXXS | C |
| 10A | [EK]LXX[LI]EX[QA] | C |
| 10B | [GN][DE]XXX[SG]X[SN] | K |
| 11A | [IV]FSXXX[KR]L | I |
| 11B | XXXSSXXS | C |

^a^ A and B in motif number indicate two different motifs which are complementary to each other and form a correlated motif pair (see Methods for further explanation).

^b^ Brackets enclose alternative residues allowed at one position, X indicates wildcard (all residues allowed).

^c^ Domain indicates domain in which motif mainly occurs (note that in a couple of cases motifs also have a few occurrences outside this domain). I = I-region, K = K-box, C = C-terminal region.

**S1.B. IMSS motif-hit positions**

| **MADS** | **Motif position** | **Motif nr. ^a^** | **MADS** | **Motif position** | **Motif nr. ^a^** |
| --- | --- | --- | --- | --- | --- |
| AG | 129 | 10B | AGL79 | 46 | 2B/1A/11A |
| AG | 138 | 3A/8B | AGL79 | 49 | 4A |
| AG | 61 | 5B/6B | AGL79 | 62 | 3B |
| AG | 63 | 2B/1A/11A | ANR1 | 103 | 9A |
| AG | 66 | 4A | ANR1 | 112 | 10B |
| AG | 77 | 8A | ANR1 | 123 | 9A |
| AGL12 | 129 | 3A/8B | ANR1 | 164 | 7A |
| PI | 100 | 9A | ANR1 | 44 | 5B/6B |
| PI | 109 | 10B | ANR1 | 46 | 2B/1A/11A |
| PI | 197 | 9A | ANR1 | 49 | 4A |
| AGL12 | 44 | 5B/6B | ANR1 | 59 | 6A |
| AGL12 | 46 | 2B/1A/11A | ANR1 | 61 | 8A |
| AGL12 | 49 | 4A | ANR1 | 62 | 3B |
| AGL13 | 110 | 10B | ANR1 | 74 | 3A |
| AGL13 | 119 | 3A/7A/8B | ANR1 | 96 | 9A |
| AGL13 | 120 | 10A | AP1 | 122 | 3A/7A/8B |
| AGL13 | 121 | 9A | AP1 | 123 | 10A |
| AGL13 | 44 | 5B/6B | AP1 | 124 | 9A |
| AGL13 | 46 | 2B/1A/11A | AP1 | 151 | 7A |
| AGL13 | 49 | 4A | AP1 | 44 | 5B/6B |
| AGL13 | 53 | 5B | AP1 | 46 | 2B/1A/11A |
| AGL14 | 121 | 3A/7A/8B | AP1 | 49 | 4A |
| AGL14 | 122 | 10A | AP1 | 62 | 3B |
| AGL14 | 123 | 9A | CAL | 126 | 9A |
| AGL14 | 179 | 9B/11B | CAL | 242 | 9A |
| AGL14 | 181 | 9B/11B | CAL | 44 | 5B/6B |
| AGL14 | 44 | 5B/6B | CAL | 46 | 2B/1A/11A |
| AGL14 | 46 | 2B/1A/11A | CAL | 49 | 4A |
| AGL14 | 49 | 4A | CAL | 62 | 3B |
| AGL14 | 54 | 9B/11B | FUL | 113 | 10B |
| AGL15 | 101 | 9A | FUL | 122 | 3A/7A/8B |
| AGL15 | 114 | 3A/7A/8B | FUL | 123 | 10A |
| AGL15 | 115 | 10A | FUL | 124 | 9A |
| AGL15 | 116 | 9A | FUL | 153 | 9A |
| AGL15 | 44 | 5B/6B | FUL | 160 | 9A |
| AGL15 | 46 | 2B/1A/11A | FUL | 44 | 5B/6B |
| AGL15 | 49 | 4A | FUL | 46 | 2B/1A/11A |
| AGL15 | 53 | 5B | FUL | 49 | 4A |
| AGL15 | 70 | 9B/11B | FUL | 62 | 3B |
| AGL15 | 96 | 9A | FUL | 66 | 9A |
| AGL16 | 110 | 10B | FUL | 75 | 8A |
| AGL16 | 119 | 3A/8B | MAF1/FLM | 49 | 4A |
| AGL16 | 121 | 9A | MAF1/FLM | 96 | 9A |
| AGL16 | 44 | 5B/6B | SEP1 | 104 | 9A |
| AGL16 | 46 | 2B/1A/11A | SEP1 | 122 | 3A/8B |
| AGL16 | 49 | 4A | SEP1 | 123 | 10A |
| AGL16 | 55 | 1B | SEP1 | 124 | 9A |
| AGL16 | 56 | 4B/5A/7B/9B/11B | SEP1 | 153 | 9A |
| AGL16 | 58 | 6A | SEP1 | 156 | 3A |
| AGL16 | 60 | 8A | SEP1 | 44 | 5B/6B |
| AGL16 | 61 | 3B | SEP1 | 46 | 2B/1A/11A |
| AGL17 | 102 | 9A | SEP1 | 49 | 4A |
| AGL17 | 120 | 3A/7A/8B | SEP1 | 56 | 4B/7B |
| AGL17 | 121 | 10A | SEP1 | 62 | 3B |
| AGL17 | 122 | 9A | SEP2 | 104 | 9A |
| AGL17 | 44 | 5B | SEP2 | 122 | 3A/8B |
| AGL17 | 46 | 1A/11A | SEP2 | 123 | 10A |
| AGL17 | 56 | 4B/7B/9B/11B | SEP2 | 124 | 9A |
| AGL17 | 58 | 6A | SEP2 | 153 | 9A |
| AGL17 | 60 | 8A | SEP2 | 44 | 5B/6B |
| AGL18 | 102 | 8A | SEP2 | 46 | 2B/1A/11A |
| AGL18 | 110 | 9A | SEP2 | 49 | 4A |
| AGL18 | 44 | 5B/6B | SEP2 | 62 | 3B |
| AGL19 | 102 | 9A | SEP3 | 107 | 9A |
| AGL19 | 120 | 3A/7A/8B | SEP3 | 125 | 3A/8B |
| AGL19 | 121 | 10A | SEP3 | 126 | 10A |
| AGL19 | 122 | 9A | SEP3 | 127 | 9A |
| AGL19 | 44 | 6B | SEP3 | 156 | 9A |
| AGL19 | 46 | 1A/11A | SEP3 | 44 | 5B/6B |
| AGL21 | 111 | 10B | SEP3 | 46 | 2B/1A/11A |
| AGL21 | 121 | 10A | SEP3 | 49 | 4A |
| AGL21 | 44 | 5B/6B | SEP3 | 56 | 4B/7B |
| AGL21 | 46 | 2B/1A/11A | SEP3 | 62 | 3B |
| AGL21 | 49 | 4A | SEP4-I | 104 | 9A |
| AGL21 | 55 | 1B | SEP4-I | 123 | 10A |
| AGL21 | 56 | 4B/5A/7B/9B/11B | SEP4-I | 171 | 9A |
| AGL21 | 58 | 6A | SEP4-I | 199 | 9B/11B |
| AGL21 | 60 | 8A | SEP4-I | 44 | 5B/6B |
| AGL21 | 61 | 3B | SEP4-I | 46 | 2B/1A/11A |
| AGL21 | 73 | 3A | SEP4-I | 49 | 4A |
| AGL24 | 112 | 10B | SHP1 | 127 | 10B |
| AGL24 | 121 | 3A/7A/8B | SHP1 | 136 | 3A/8B |
| AGL24 | 123 | 9A | SHP1 | 172 | 7A |
| AGL24 | 152 | 9A | SHP1 | 174 | 9A |
| AGL24 | 169 | 10A | SHP1 | 59 | 5B/6B |
| AGL24 | 192 | 9B/11B | SHP1 | 61 | 2B/1A/11A |
| AGL24 | 44 | 5B/6B | SHP1 | 64 | 4A |
| AGL24 | 46 | 2B/1A/11A | SHP2 | 127 | 10B |
| AGL24 | 49 | 4A | SHP2 | 136 | 3A/8B |
| AGL24 | 55 | 1B | SHP2 | 172 | 7A |
| AGL24 | 56 | 5A | SHP2 | 174 | 9A |
| AGL25/FLC | 96 | 9A | SHP2 | 208 | 9B/11B |
| AGL31 | 145 | 9A | SHP2 | 59 | 5B/6B |
| AGL31 | 62 | 3B | SHP2 | 61 | 2B/1A/11A |
| AGL31 | 96 | 9A | SHP2 | 64 | 4A |
| AGL32 | 121 | 10A | SOC1 | 103 | 9A |
| AGL32 | 230 | 9A | SOC1 | 121 | 3A/7A/8B |
| AGL32 | 44 | 5B/6B | SOC1 | 122 | 10A |
| AGL32 | 46 | 2B/1A/11A | SOC1 | 123 | 9A |
| AGL32 | 63 | 3B | SOC1 | 133 | 8A |
| AGL42 | 103 | 9A | SOC1 | 189 | 10B |
| AGL42 | 121 | 7A/8B | SOC1 | 190 | 9B/11B |
| AGL42 | 123 | 9A | SOC1 | 195 | 2A |
| AGL42 | 44 | 5B/6B | SOC1 | 44 | 5B/6B |
| AGL42 | 46 | 2B/1A/11A | SOC1 | 46 | 2B/1A/11A |
| AGL42 | 49 | 4A | SOC1 | 49 | 4A |
| AGL42 | 53 | 5B | SOC1 | 55 | 1B |
| AGL42 | 55 | 1B | SOC1 | 56 | 5A |
| AGL42 | 56 | 5A | SOC1 | 61 | 3B |
| AGL42 | 61 | 3B | STK | 112 | 10B |
| AGL6 | 120 | 3A/7A/8B | STK | 121 | 3A |
| AGL6 | 121 | 10A | STK | 44 | 5B/6B |
| AGL6 | 122 | 9A | STK | 46 | 2B/1A/11A |
| AGL63 | 46 | 1A/11A | STK | 49 | 4A |
| AGL6 | 44 | 5B/6B | SVP2 | 113 | 7A/8B |
| AGL6 | 46 | 2B/1A/11A | SVP2 | 121 | 3A/7A/8B |
| AGL6 | 49 | 4A | SVP2 | 122 | 10A |
| AGL68 | 145 | 9A | SVP2 | 123 | 9A |
| AGL68 | 181 | 9B/11B | SVP2 | 150 | 7A |
| AGL68 | 54 | 9B/11B | SVP2 | 152 | 9A |
| AGL69 | 54 | 9B/11B | SVP2 | 44 | 5B/6B |
| AGL70 | 145 | 9A | SVP2 | 46 | 2B/1A/11A |
| AGL70 | 49 | 4A | SVP2 | 49 | 4A |
| AGL70 | 62 | 3B | SVP2 | 55 | 1B |
| AGL70 | 96 | 9A | SVP2 | 56 | 4B/7B |
| AGL71 | 104 | 9A | SVP2 | 58 | 6A |
| AGL71 | 122 | 7A | SVP2 | 60 | 8A |
| AGL71 | 46 | 2B/1A/11A | SVP2 | 83 | 3A/7A |
| AGL71 | 61 | 3B | SVP1 | 114 | 7A/8B |
| AGL72 | 148 | 7A | SVP1 | 122 | 3A/7A/8B |
| AGL72 | 46 | 2B/1A/11A | SVP1 | 123 | 10A |
| AGL72 | 49 | 4A | SVP1 | 124 | 9A |
| AGL74 | 157 | 10B | SVP1 | 151 | 7A |
| AGL74 | 158 | 9B/11B | SVP1 | 153 | 9A |
| AGL74 | 159 | 9B/11B | SVP1 | 224 | 9B/11B |
| AGL74 | 161 | 6A | SVP1 | 45 | 5B/6B |
| AGL74 | 199 | 2A | SVP1 | 47 | 2B/1A/11A |
| AGL74 | 217 | 9B/11B | SVP1 | 50 | 4A |
| AGL79 | 104 | 9A | SVP1 | 56 | 1B |
| AGL79 | 113 | 10B | SVP1 | 57 | 4B/7B |
| AGL79 | 124 | 9A | SVP1 | 59 | 6A |
| AGL79 | 153 | 9A | SVP1 | 61 | 8A |
| AGL79 | 44 | 5B/6B | SVP1 | 84 | 3A/7A |

^a^ A and B indicate two different motifs which are complementary to each other and form a correlated motif pair (see Methods for further explanation).

S1.C. IMSS motif-hits

| **MADS** | **Position** | **Motif-hit** | **MADS** | **Position** | **Motif-hit** |
| --- | --- | --- | --- | --- | --- |
| AG | 129 | "GEtigSmS" | AGL79 | 44 | "LiVFSpkG" |
| AG | 138 | "KELrnLEg" | AGL79 | 46 | "vFSpkGkL" |
| AG | 61 | "LiVFSsrG" | AGL79 | 46 | "VFSpkgKL" |
| AG | 63 | "vFSsrGrL" | AGL79 | 49 | "pkGKLFEy" |
| AG | 63 | "VFSsrgRL" | AGL79 | 62 | "MeriLDRY" |
| AG | 66 | "srGRLYEy" | ANR1 | 103 | "LQechrkL" |
| AG | 77 | "SvKgtieR" | ANR1 | 112 | "GEelsGmN" |
| AGL12 | 129 | "EELllLEk" | ANR1 | 123 | "LQnledqL" |
| PI | 100 | "LQlelrhL" | ANR1 | 164 | "hELQnivd" |
| PI | 109 | "GEdiqSlN" | ANR1 | 44 | "ViIFSstG" |
| PI | 197 | "LQekimsL" | ANR1 | 46 | "iFSstGkL" |
| AGL12 | 44 | "VvIFSpqG" | ANR1 | 46 | "IFSstgKL" |
| AGL12 | 46 | "iFSpqGkL" | ANR1 | 49 | "stGKLYDy" |
| AGL12 | 46 | "IFSpqgKL" | ANR1 | 59 | "NSSmKtiI" |
| AGL12 | 49 | "pqGKLFEl" | ANR1 | 61 | "SmKtiieR" |
| AGL13 | 110 | "GEdleGmS" | ANR1 | 62 | "MktiIERY" |
| AGL13 | 119 | "kELQtler" | ANR1 | 74 | "EEQhqLLn" |
| AGL13 | 119 | "KELqtLEr" | ANR1 | 96 | "LQqqlqyL" |
| AGL13 | 120 | "ELqtLErQ" | AP1 | 122 | "kELQnleq" |
| AGL13 | 121 | "LQtlerqL" | AP1 | 122 | "KELqnLEq" |
| AGL13 | 44 | "LiIFStgG" | AP1 | 123 | "ELqnLEqQ" |
| AGL13 | 46 | "iFStgGkL" | AP1 | 124 | "LQnleqqL" |
| AGL13 | 46 | "IFStggKL" | AP1 | 151 | "nELQkkek" |
| AGL13 | 49 | "tgGKLYEf" | AP1 | 44 | "LvVFShkG" |
| AGL13 | 53 | "LyEFSnvG" | AP1 | 46 | "vFShkGkL" |
| AGL14 | 121 | "eELQqlen" | AP1 | 46 | "VFShkgKL" |
| AGL14 | 121 | "EELqqLEn" | AP1 | 49 | "hkGKLFEy" |
| AGL14 | 122 | "ELqqLEnQ" | AP1 | 62 | "MekiLERY" |
| AGL14 | 123 | "LQqlenqL" | CAL | 126 | "LQnleqqL" |
| AGL14 | 179 | "griSSssS" | CAL | 242 | "LEpiynyL" |
| AGL14 | 181 | "issSSstS" | CAL | 44 | "LiVFShkG" |
| AGL14 | 44 | "LiIFSprG" | CAL | 46 | "vFShkGkL" |
| AGL14 | 46 | "iFSprGkL" | CAL | 46 | "VFShkgKL" |
| AGL14 | 46 | "IFSprgKL" | CAL | 49 | "hkGKLFEy" |
| AGL14 | 49 | "prGKLYEf" | CAL | 62 | "MekvLERY" |
| AGL14 | 54 | "yefSSssS" | FUL | 113 | "GEdldSlS" |
| AGL15 | 101 | "LQlqgkgL" | FUL | 122 | "kELQsleh" |
| AGL15 | 114 | "kELQsleq" | FUL | 122 | "KELqsLEh" |
| AGL15 | 114 | "KELqsLEq" | FUL | 123 | "ELqsLEhQ" |
| AGL15 | 115 | "ELqsLEqQ" | FUL | 124 | "LQslehqL" |
| AGL15 | 116 | "LQsleqqL" | FUL | 153 | "LQkkdkaL" |
| AGL15 | 44 | "ViVFSksG" | FUL | 160 | "LQdhnnsL" |
| AGL15 | 46 | "vFSksGkL" | FUL | 44 | "LiVFSskG" |
| AGL15 | 46 | "VFSksgKL" | FUL | 46 | "vFSskGkL" |
| AGL15 | 49 | "ksGKLFEy" | FUL | 46 | "VFSskgKL" |
| AGL15 | 53 | "LfEYSstG" | FUL | 49 | "skGKLFEy" |
| AGL15 | 70 | "nhqSSsaS" | FUL | 62 | "MeriLERY" |
| AGL15 | 96 | "LQekhlqL" | FUL | 66 | "LErydryL" |
| AGL16 | 110 | "GEelsGlS" | FUL | 75 | "SdKqlvgR" |
| AGL16 | 119 | "EALqnLEn" | MAF1/FLM | 49 | "asGKLYDs" |
| AGL16 | 121 | "LQnlenqL" | MAF1/FLM | 96 | "LEtvqskL" |
| AGL16 | 44 | "ViIFSstG" | SEP1 | 104 | "LQrqqrnL" |
| AGL16 | 46 | "iFSstGrL" | SEP1 | 122 | "KELeqLEr" |
| AGL16 | 46 | "IFSstgRL" | SEP1 | 123 | "ELeqLErQ" |
| AGL16 | 49 | "stGRLYDf" | SEP1 | 124 | "LEqlerqL" |
| AGL16 | 55 | "DFsSSsMk" | SEP1 | 153 | "LQnkeqmL" |
| AGL16 | 56 | "fssSSmkS" | SEP1 | 156 | "KEQmlLEt" |
| AGL16 | 56 | "FsSSSmKs" | SEP1 | 44 | "LiIFSnrG" |
| AGL16 | 56 | "FSSSsMks" | SEP1 | 46 | "iFSnrGkL" |
| AGL16 | 58 | "SSSmKsvI" | SEP1 | 46 | "IFSnrgKL" |
| AGL16 | 60 | "SmKsvieR" | SEP1 | 49 | "nrGKLYEf" |
| AGL16 | 61 | "MksvIERY" | SEP1 | 56 | "FcSSSnMl" |
| AGL17 | 102 | "LQenyrqL" | SEP1 | 62 | "MlktLDRY" |
| AGL17 | 120 | "kELQnies" | SEP2 | 104 | "LQrqqrnL" |
| AGL17 | 120 | "KELqnIEs" | SEP2 | 122 | "KELeqLEr" |
| AGL17 | 121 | "ELqnIEsQ" | SEP2 | 123 | "ELeqLErQ" |
| AGL17 | 122 | "LQniesqL" | SEP2 | 124 | "LEqlerqL" |
| AGL17 | 44 | "LiIFSntD" | SEP2 | 153 | "LQgkehiL" |
| AGL17 | 46 | "IFSntdKL" | SEP2 | 44 | "LiVFSnrG" |
| AGL17 | 56 | "fasSSvkS" | SEP2 | 46 | "vFSnrGkL" |
| AGL17 | 56 | "FaSSSvKs" | SEP2 | 46 | "VFSnrgKL" |
| AGL17 | 58 | "SSSvKstI" | SEP2 | 49 | "nrGKLYEf" |
| AGL17 | 60 | "SvKstieR" | SEP2 | 62 | "MlktLERY" |
| AGL18 | 102 | "SmKgeleR" | SEP3 | 107 | "LQrtqrnL" |
| AGL18 | 110 | "LQlaierL" | SEP3 | 125 | "KELesLEr" |
| AGL18 | 44 | "LiIFSstG" | SEP3 | 126 | "ELesLErQ" |
| AGL19 | 102 | "LEiskrkL" | SEP3 | 127 | "LEslerqL" |
| AGL19 | 120 | "eELQqlen" | SEP3 | 156 | "LQskermL" |
| AGL19 | 120 | "EELqqLEn" | SEP3 | 44 | "LiIFSnrG" |
| AGL19 | 121 | "ELqqLEnQ" | SEP3 | 46 | "iFSnrGkL" |
| AGL19 | 122 | "LQqlenqL" | SEP3 | 46 | "IFSnrgKL" |
| AGL19 | 44 | "LvIFSprS" | SEP3 | 49 | "nrGKLYEf" |
| AGL19 | 46 | "IFSprsKL" | SEP3 | 56 | "FcSSSsMl" |
| AGL21 | 111 | "GEqlnGlS" | SEP3 | 62 | "MlrtLERY" |
| AGL21 | 121 | "ELnsLEnQ" | SEP4-I | 104 | "LQhsqrhL" |
| AGL21 | 44 | "LiIFSstG" | SEP4-I | 123 | "ELehLErQ" |
| AGL21 | 46 | "iFSstGkL" | SEP4-I | 171 | "LEdsdaaL" |
| AGL21 | 46 | "IFSstgKL" | SEP4-I | 199 | "qgmSSyqS" |
| AGL21 | 49 | "stGKLYDf" | SEP4-I | 44 | "LlIFSnrG" |
| AGL21 | 55 | "DFaSSsMk" | SEP4-I | 46 | "iFSnrGkL" |
| AGL21 | 56 | "fasSSmkS" | SEP4-I | 46 | "IFSnrgKL" |
| AGL21 | 56 | "FaSSSmKs" | SEP4-I | 49 | "nrGKLYEf" |
| AGL21 | 56 | "FASSsMks" | SHP1 | 127 | "GEslgSlN" |
| AGL21 | 58 | "SSSmKsvI" | SHP1 | 136 | "KELknLEg" |
| AGL21 | 60 | "SmKsvidR" | SHP1 | 172 | "mELQhnnm" |
| AGL21 | 61 | "MksvIDRY" | SHP1 | 174 | "LQhnnmyL" |
| AGL21 | 73 | "IEQqqLLn" | SHP1 | 59 | "LvIFStrG" |
| AGL24 | 112 | "GEdldGlN" | SHP1 | 61 | "iFStrGrL" |
| AGL24 | 121 | "eELQrlek" | SHP1 | 61 | "IFStrgRL" |
| AGL24 | 121 | "EELqrLEk" | SHP1 | 64 | "trGRLYEy" |
| AGL24 | 123 | "LQrleklL" | SHP2 | 127 | "GEslgSlN" |
| AGL24 | 152 | "LEkrgseL" | SHP2 | 136 | "KELknLEs" |
| AGL24 | 169 | "KLetLErA" | SHP2 | 172 | "iELQndnm" |
| AGL24 | 192 | "tnvSSydS" | SHP2 | 174 | "LQndnmyL" |
| AGL24 | 44 | "LiIFSatG" | SHP2 | 208 | "gvtSShqS" |
| AGL24 | 46 | "iFSatGkL" | SHP2 | 59 | "LvIFStrG" |
| AGL24 | 46 | "IFSatgKL" | SHP2 | 61 | "iFStrGrL" |
| AGL24 | 49 | "atGKLFEf" | SHP2 | 61 | "IFStrgRL" |
| AGL24 | 55 | "EFsSSrMr" | SHP2 | 64 | "trGRLYEy" |
| AGL24 | 56 | "FSSSrMrd" | SOC1 | 103 | "LEaskrkL" |
| AGL25/FLC | 96 | "LElvdskL" | SOC1 | 121 | "eELQqieq" |
| AGL31 | 145 | "LQktenlL" | SOC1 | 121 | "EELqqIEq" |
| AGL31 | 62 | "MskiIDRY" | SOC1 | 122 | "ELqqIEqQ" |
| AGL31 | 96 | "LEivqskL" | SOC1 | 123 | "LQqieqqL" |
| AGL32 | 121 | "ELdgLErQ" | SOC1 | 133 | "SvKciraR" |
| AGL32 | 230 | "LQlaqpnL" | SOC1 | 189 | "GDeesSpS" |
| AGL32 | 44 | "LiVFSatG" | SOC1 | 190 | "deeSSpsS" |
| AGL32 | 46 | "vFSatGkL" | SOC1 | 195 | "PSSeVeTq" |
| AGL32 | 46 | "VFSatgKL" | SOC1 | 44 | "LiIFSpkG" |
| AGL32 | 63 | "MpqlIDRY" | SOC1 | 46 | "iFSpkGkL" |
| AGL42 | 103 | "LEfhkrkL" | SOC1 | 46 | "IFSpkgKL" |
| AGL42 | 121 | "eELQeids" | SOC1 | 49 | "pkGKLYEf" |
| AGL42 | 121 | "EELqeIDs" | SOC1 | 55 | "EFaSSnMq" |
| AGL42 | 123 | "LQeidsqL" | SOC1 | 56 | "FASSnMqd" |
| AGL42 | 44 | "LiIFSqrG" | SOC1 | 61 | "MqdtIDRY" |
| AGL42 | 46 | "iFSqrGrL" | STK | 112 | "GDslsSlS" |
| AGL42 | 46 | "IFSqrgRL" | STK | 121 | "KELkqVEn" |
| AGL42 | 49 | "qrGRLYEf" | STK | 44 | "LiVFStrG" |
| AGL42 | 53 | "LyEFSssD" | STK | 46 | "vFStrGrL" |
| AGL42 | 55 | "EFsSSdMq" | STK | 46 | "VFStrgRL" |
| AGL42 | 56 | "FSSSdMqk" | STK | 49 | "trGRLYEy" |
| AGL42 | 61 | "MqktIERY" | SVP2 | 113 | "eELQgldi" |
| AGL6 | 120 | "kELQaler" | SVP2 | 113 | "EELqgLDi" |
| AGL6 | 120 | "KELqaLEr" | SVP2 | 121 | "eELQqlek" |
| AGL6 | 121 | "ELqaLErQ" | SVP2 | 121 | "EELqqLEk" |
| AGL6 | 122 | "LQalerqL" | SVP2 | 122 | "ELqqLEkA" |
| AGL63 | 46 | "IFShsnRL" | SVP2 | 123 | "LQqlekaL" |
| AGL6 | 44 | "LiIFSsrG" | SVP2 | 150 | "sELQkkgm" |
| AGL6 | 46 | "iFSsrGkL" | SVP2 | 152 | "LQkkgmqL" |
| AGL6 | 46 | "IFSsrgKL" | SVP2 | 44 | "LiIFSstG" |
| AGL6 | 49 | "srGKLYEf" | SVP2 | 46 | "iFSstGkL" |
| AGL68 | 145 | "LQdkeklL" | SVP2 | 46 | "IFSstgKL" |
| AGL68 | 181 | "penSSghS" | SVP2 | 49 | "stGKLFEf" |
| AGL68 | 54 | "ynsSSgdS" | SVP2 | 55 | "EFcSSsMk" |
| AGL69 | 54 | "ysfSSgdS" | SVP2 | 56 | "FcSSSmKe" |
| AGL70 | 145 | "LQereklL" | SVP2 | 58 | "SSSmKevL" |
| AGL70 | 49 | "gsGKLYDs" | SVP2 | 60 | "SmKevleR" |
| AGL70 | 62 | "MskiIDRY" | SVP2 | 83 | "lELQlven" |
| AGL70 | 96 | "LEivqskL" | SVP2 | 83 | "LELqlVEn" |
| AGL71 | 104 | "LEvhhrkL" | SVPl | 114 | "eELQgldi" |
| AGL71 | 122 | "tELQeidt" | SVPl | 114 | "EELqgLDi" |
| AGL71 | 46 | "vFSqsGrL" | SVPl | 122 | "eELQqlek" |
| AGL71 | 46 | "VFSqsgRL" | SVPl | 122 | "EELqqLEk" |
| AGL71 | 61 | "MekiIDRY" | SVPl | 123 | "ELqqLEkA" |
| AGL72 | 148 | "dELQklka" | SVPl | 124 | "LQqlekaL" |
| AGL72 | 46 | "iFSqkGrL" | SVPl | 151 | "sELQkkgm" |
| AGL72 | 46 | "IFSqkgRL" | SVPl | 153 | "LQkkgmqL" |
| AGL72 | 49 | "qkGRLYEf" | SVPl | 224 | "dseSSdtS" |
| AGL74 | 157 | "NDsgsSsS" | SVPl | 45 | "LiIFSstG" |
| AGL74 | 158 | "dsgSSssS" | SVPl | 47 | "iFSstGkL" |
| AGL74 | 159 | "sgsSSssS" | SVPl | 47 | "IFSstgKL" |
| AGL74 | 161 | "SSSsSsqI" | SVPl | 50 | "stGKLFEf" |
| AGL74 | 199 | "PSSqVtTc" | SVPl | 56 | "EFcSSsMk" |
| AGL74 | 217 | "sesSSdqS" | SVPl | 57 | "FcSSSmKe" |
| AGL79 | 104 | "LQrslrhL" | SVPl | 59 | "SSSmKevL" |
| AGL79 | 113 | "GEevdGlS" | SVPl | 61 | "SmKevleR" |
| AGL79 | 124 | "LQgvemqL" | SVPl | 84 | "lELQlven" |
| AGL79 | 153 | "LQkkekeL" | SVPl | 84 | "LELqlVEn" |
