# Supplementary material for: Sequence Motifs in MADS Transcription Factors Responsible for Specificity and Diversification of Protein-Protein Interaction
Source: PLoS Comput Biol. 2010 Nov 24;6(11):e1001017. doi: 10.1371/journal.pcbi.1001017 (PMC2991254; doi:10.1371/journal.pcbi.1001017)
Supplement: Table S2 — Interaction data from additional species. (0.20 MB DOC) [file pcbi.1001017.s004.doc]

**Table S2. Interaction data from additional species**

| **Interacting MADS pair^a^** | | U**niprot identifiers** | | **Interacting MADS pair^a^** | | U**niprot identifiers** | |
| --- | --- | --- | --- | --- | --- | --- | --- |
| pMADS12 | FBP6 | Q6UGQ8 | Q08711 | LpMADS1 | LpMADS10 | Q84UA3 | Q45V73 |
| pMADS12 | FBP11 | Q6UGQ8 | Q40882 | LpMADS2 | LpMADS2 | Q84UA2 | Q84UA2 |
| FBP4 | FBP21 | Q9ATF3 | Q9ATE8 | LpMADS2 | LpMADS10 | Q84UA2 | Q45V73 |
| FBP5 | FBP13 | Q9ATF2 | Q9ATF0 | LpMADS3 | LpMADS10 | Q84UA1 | Q45V73 |
| FBP5 | FBP21 | Q9ATF2 | Q9ATE8 | LpMADS1 | AG | Q84UA3 | 42566942 |
| FBP5 | UNS | Q9ATF2 | Q9ATE9 | LpMADS1 | SHP1 | Q84UA3 | 862640 |
| FBP5 | FBP22 | Q9ATF2 | Q9ATE7 | LpMADS1 | SHP2 | Q84UA3 | 166596 |
| FBP5 | FBP29 | Q9ATF2 | Q9ATE2 | LpMADS1 | SEP4-I | Q84UA3 | 1737495 |
| FBP5 | FBP28 | Q9ATF2 | Q9ATE3 | LpMADS1 | SEP4-II | Q84UA3 | 32402422 |
| FBP5 | FBP6 | Q9ATF2 | Q08711 | LpMADS1 | SEP2 | Q84UA3 | 15232883 |
| FBP5 | pMADS3 | Q9ATF2 | Q40885 | LpMADS1 | FUL | Q84UA3 | 1004365 |
| FBP5 | FBP11 | Q9ATF2 | Q40882 | LpMADS1 | STK | Q84UA3 | 166588 |
| FBP5 | FBP7 | Q9ATF2 | Q43616 | LpMADS1 | AGL13 | Q84UA3 | 15233045 |
| FBP9 | FBP21 | Q9ATF1 | Q9ATE8 | LpMADS1 | AGL14 | Q84UA3 | 15234342 |
| FBP9 | UNS | Q9ATF1 | Q9ATE9 | LpMADS1 | AGL15 | Q84UA3 | 15240706 |
| FBP9 | FBP22 | Q9ATF1 | Q9ATE7 | LpMADS1 | SOC1 | Q84UA3 | 15225534 |
| FBP9 | FBP28 | Q9ATF1 | Q9ATE3 | LpMADS1 | AGL21 | Q84UA3 | 15235748 |
| FBP6 | FBP9 | Q08711 | Q9ATF1 | LpMADS1 | SVP | Q84UA3 | 99999999 |
| FBP9 | pMADS3 | Q9ATF1 | Q40885 | LpMADS1 | AGL24 | Q84UA3 | 15233857 |
| FBP9 | FBP11 | Q9ATF1 | Q40882 | LpMADS1 | AGL42 | Q84UA3 | 32402390 |
| FBP9 | FBP7 | Q9ATF1 | Q43616 | LpMADS2 | AG | Q84UA2 | 42566942 |
| FBP13 | FBP13 | Q9ATF0 | Q9ATF0 | LpMADS2 | SHP1 | Q84UA2 | 862640 |
| FBP13 | FBP21 | Q9ATF0 | Q9ATE8 | LpMADS2 | SHP2 | Q84UA2 | 166596 |
| FBP13 | UNS | Q9ATF0 | Q9ATE9 | LpMADS2 | SEP4-I | Q84UA2 | 1737495 |
| FBP13 | FBP22 | Q9ATF0 | Q9ATE7 | LpMADS2 | SEP4-II | Q84UA2 | 32402422 |
| FBP13 | FBP29 | Q9ATF0 | Q9ATE2 | LpMADS2 | SEP2 | Q84UA2 | 15232883 |
| FBP13 | FBP28 | Q9ATF0 | Q9ATE3 | LpMADS2 | FUL | Q84UA2 | 1004365 |
| pMADS12 | FBP21 | Q6UGQ8 | Q9ATE8 | LpMADS2 | STK | Q84UA2 | 166588 |
| FBP21 | FBP29 | Q9ATE8 | Q9ATE2 | LpMADS2 | AGL13 | Q84UA2 | 15233045 |
| FBP2 | FBP21 | Q03489 | Q9ATE8 | LpMADS2 | AGL14 | Q84UA2 | 15234342 |
| pMADS12 | UNS | Q6UGQ8 | Q9ATE9 | LpMADS2 | AGL15 | Q84UA2 | 15240706 |
| UNS | FBP25 | Q9ATE9 | Q9ATE4 | LpMADS2 | SOC1 | Q84UA2 | 15225534 |
| UNS | FBP23 | Q9ATE9 | Q9ATE6 | LpMADS2 | AGL21 | Q84UA2 | 15235748 |
| FBP2 | UNS | Q03489 | Q9ATE9 | LpMADS2 | SVP | Q84UA2 | 99999999 |
| FBP23 | FBP29 | Q9ATE6 | Q9ATE2 | LpMADS2 | AGL24 | Q84UA2 | 15233857 |
| FBP23 | FBP28 | Q9ATE6 | Q9ATE3 | LpMADS2 | AGL42 | Q84UA2 | 32402390 |
| FBP6 | FBP23 | Q08711 | Q9ATE6 | LpMADS10 | SHP1 | Q45V73 | 862640 |
| FBP23 | pMADS3 | Q9ATE6 | Q40885 | LpMADS10 | SHP2 | Q45V73 | 166596 |
| FBP11 | FBP23 | Q40882 | Q9ATE6 | LpMADS10 | SEP2 | Q45V73 | 15232883 |
| FBP22 | FBP29 | Q9ATE7 | Q9ATE2 | LpMADS10 | FUL | Q45V73 | 1004365 |
| FBP2 | FBP22 | Q03489 | Q9ATE7 | LpMADS10 | STK | Q45V73 | 166588 |
| FBP2 | FBP29 | Q03489 | Q9ATE2 | LpMADS10 | AGL12 | Q45V73 | 18409883 |
| FBP28 | FBP29 | Q9ATE3 | Q9ATE2 | LpMADS10 | AGL13 | Q45V73 | 15233045 |
| FBP2 | FBP28 | Q03489 | Q9ATE3 | LpMADS10 | AGL14 | Q45V73 | 15234342 |
| FBP21 | pMADS4 | Q9ATE8 | Q9MB91 | LpMADS10 | AGL15 | Q45V73 | 15240706 |
| FBP22 | pMADS4 | Q9ATE7 | Q9MB91 | LpMADS10 | SOC1 | Q45V73 | 15225534 |
| FBP28 | pMADS4 | Q9ATE3 | Q9MB91 | LpMADS10 | AGL21 | Q45V73 | 15235748 |
| FBP2 | FBP6 | Q03489 | Q08711 | LpMADS10 | SVP | Q45V73 | 99999999 |
| pMADS1 | FBP1 | Q07472 | Q6QNL4 | LpMADS10 | AGL42 | Q45V73 | 32402390 |
| FBP2 | pMADS3 | Q03489 | Q40885 | LpMADS10 | AGL71 | Q45V73 | 32402406 |
| FBP2 | FBP11 | Q03489 | Q40882 | PTM5 | PTM5 | Q7Y137 | Q7Y137 |
| pMADS1 | pMADS2 | Q07472 | Q07474 | TaVRT-2 | TaVRT-1 | Q4U0F1 | Q84LI3 |
| FBP2 | FBP7 | Q03489 | Q43616 | TaVRT-2 | TaVRT-2 | Q4U0F1 | Q4U0F1 |
| DEF | GLO | P23706 | Q03378 | DAL11 | DAL11 | Q9SEE3 | Q9SEE3 |
| FAR | DEFH200 | Q9XFM8 | Q38733 | DAL11 | DAL13 | Q9SEE3 | Q9SED8 |
| FAR | DEFH49 | Q9XFM8 | Q38734 | DAL11 | AP3 | Q9SEE3 | 15232493 |
| PLE | DEFH200 | Q41195 | Q38733 | DAL13 | DAL13 | Q9SED8 | Q9SED8 |
| PLE | DEFH72 | Q41195 | Q38735 | DAL13 | AP3 | Q9SED8 | 15232493 |
| PLE | DEFH49 | Q41195 | Q38734 | OsMADS18 | OsMADS24 | Q0D4T4 | Q9SAR1 |
| SQUA | DEFH200 | Q38742 | Q38733 | OsMADS18 | OsMADS45 | Q0D4T4 | Q0J466 |
| SQUA | DEFH72 | Q38742 | Q38735 | OsMADS18 | OsMADS6 | Q0D4T4 | Q6EU39 |
| CDM44 | CDM8 | Q84LD3 | Q84LD4 | OsMADS18 | OsMADS47 | Q0D4T4 | Q5K4R0 |
| CDM44 | CDM41 | Q84LD3 | Q84LD5 | OsMADS18 | SEP1 | Q0D4T4 | 18417790 |
| CDM44 | CDM19 | Q84LD3 | Q84LC7 | OsMADS18 | SEP3 | Q0D4T4 | 2345158 |
| CDM115 | CDM86 | Q84LD0 | Q84LC9 | WSEP-A | WAP3/WPI-2 | A7BJ55 | Q76DP8 |
| CDM19 | CDM86 | Q84LC7 | Q84LC9 | WSEP-A | WAG-1 | A7BJ55 | Q70JR2 |
| GAGA1 | GRCD1 | Q9ZS30 | Q9FST1 | WSEP-A | WAG-2 | A7BJ55 | Q8GTP4 |
| GAGA2 | GRCD1 | Q9ZS29 | Q9FST1 | WSEP-A | WLHS1-B | A7BJ55 | A7BJ59 |
| LRDEF | LRGLOB | Q8LT10 | Q8LT08 | WSEP-A | WLHS1-D | A7BJ55 | Q1G169 |
| TDR4 | TAGL2 | Q8H284 | Q8H278 | WSEP-A | WSEP-A | A7BJ55 | A7BJ55 |
| TDR4 | TAG1 | Q8H284 | Q40168 | WSEP-A | WSEP-B | A7BJ55 | A7BJ56 |
| TAGL2 | TAGL1 | Q8H278 | Q8H281 | WSEP-A | WSEP-D | A7BJ55 | A7BJ57 |
| TAGL2 | TAGL11 | Q8H278 | Q8H280 | WSEP-B | WAP3/WPI-2 | A7BJ56 | Q76DP8 |
| NtMADS11 | NtMADS4 | Q8GT99 | Q9LM09 | WSEP-B | WAG-1 | A7BJ56 | Q70JR2 |
| OsMADS16 | OsMADS4 | Q944S9 | Q40703 | WSEP-B | WAG-2 | A7BJ56 | Q8GTP4 |
| OsMADS18 | OsMADS47 | Q0D4T4 | Q5K4R0 | WSEP-B | WLHS1-B | A7BJ56 | A7BJ59 |
| OsMADS14 | OsMADS1 | Q10CQ1 | Q10PZ9 | WSEP-B | WLHS1-D | A7BJ56 | Q1G169 |
| OsMADS14 | OsMADS6 | Q10CQ1 | Q6EU39 | WSEP-B | WSEP-A | A7BJ56 | A7BJ55 |
| OsMADS15 | OsMADS1 | Q6Q9I2 | Q10PZ9 | WSEP-B | WSEP-B | A7BJ56 | A7BJ56 |
| OsMADS15 | OsMADS6 | Q6Q9I2 | Q6EU39 | WSEP-B | WSEP-D | A7BJ56 | A7BJ57 |
| OsMADS13 | OsMADS24 | Q2QW53 | Q9SAR1 | WSEP-D | WAP3/WPI-2 | A7BJ57 | Q76DP8 |
| OsMADS13 | OsMADS45 | Q2QW53 | Q0J466 | WSEP-D | WAG-1 | A7BJ57 | Q70JR2 |
| OsMADS6 | OsMADS24 | Q6EU39 | Q9SAR1 | WSEP-D | WAG-2 | A7BJ57 | Q8GTP4 |
| OsMADS6 | OsMADS45 | Q6EU39 | Q0J466 | WSEP-D | WLHS1-B | A7BJ57 | A7BJ59 |
| OsMADS6 | OsMADS18 | Q6EU39 | Q0D4T4 | WSEP-D | WLHS1-D | A7BJ57 | Q1G169 |
| FBP2 | FBP26 | Q03489 | Q6UGS3 | WSEP-D | WSEP-A | A7BJ57 | A7BJ55 |
| FBP6 | FBP26 | Q08711 | Q6UGS3 | WSEP-D | WSEP-B | A7BJ57 | A7BJ56 |
| FBP13 | FBP26 | Q9ATF0 | Q6UGS3 | WSEP-D | WSEP-D | A7BJ57 | A7BJ57 |
| FBP21 | FBP26 | Q9ATE8 | Q6UGS3 | WLHS1-B | WAP3/WPI-2 | A7BJ59 | Q76DP8 |
| FBP22 | FBP26 | Q9ATE7 | Q6UGS3 | WLHS1-B | WLHS1-B | A7BJ59 | A7BJ59 |
| FBP23 | FBP26 | Q9ATE6 | Q6UGS3 | WLHS1-B | WLHS1-D | A7BJ59 | Q1G169 |
| ZmMADS2 | ZmMADS2 | Q84KZ4 | Q84KZ4 | WLHS1-D | WAP3/WPI-2 | Q1G169 | Q76DP8 |
| LpMADS1 | LpMADS1 | Q84UA3 | Q84UA3 | WLHS1-D | WLHS1-B | Q1G169 | A7BJ59 |
| LpMADS1 | LpMADS2 | Q84UA3 | Q84UA2 | WLHS1-D | WLHS1-D | Q1G169 | Q1G169 |

^a^Data obtained from the following papers:

1. de Folter S, Immink RGH, Kieffer M, Parenicova L, Henz SR, et al. (2005) Comprehensive interaction map of the Arabidopsis MADS box transcription factors. Plant Cell 17: 1424-1433.

*2. Ciannamea S, Busscher-Lange J, de Folter S, Angenent GC, Immink RGH (2006) Characterization of the vernalization response in Lolium perenne by a cDNA microarray approach. Plant and Cell Physiology 47: 481-492.*

*3. Cseke LJ, Ravinder N, Pandey AK, Podila GK (2007) Identification of PTM5 protein interaction partners, a MADS-box gene involved in aspen tree vegetative development. Gene 391: 209-222.*

*4. Fornara F, Parenicova L, Falasca G, Pelucchi N, Masiero S, et al. (2004) Functional characterization of OsMADS18, a member of the AP1/SQUA subfamily of MADS box genes. Plant Physiology 135: 2207-2219.*

*5. Kane NA, Danyluk J, Tardif G, Ouellet F, Laliberte J-F, et al. (2005) TaVRT-2, a Member of the StMADS-11 Clade of Flowering Repressors, Is Regulated by Vernalization and Photoperiod in Wheat. Plant Physiol 138: 2354-2363.*

*6. Shan HY, Su KM, Lu WL, Kong HZ, Chen ZD, et al. (2006) Conservation and divergence of candidate class B genes in Akebia trifoliata (Lardizabalaceae). Development Genes and Evolution 216: 785-795.*

*7. Shitsukawa N, Tahira C, Kassai KI, Hirabayashi C, Shimizu T, et al. (2007) Genetic and epigenetic alteration among three homoeologous genes of a class E MADS box gene in hexaploid wheat. Plant Cell 19: 1723-1737.*

*8. Sundstrom J, Engstrom P (2002) Conifer reproductive development involves B-type MADS-box genes with distinct and different activities in male organ primordia. Plant Journal 31: 161-169.*
