# Supplementary material for: Sequence Motifs in MADS Transcription Factors Responsible for Specificity and Diversification of Protein-Protein Interaction
Source: PLoS Comput Biol. 2010 Nov 24;6(11):e1001017. doi: 10.1371/journal.pcbi.1001017 (PMC2991254; doi:10.1371/journal.pcbi.1001017)
Supplement: Table S3 — Prediction of large-scale MADS interaction data. (0.04 MB DOC) [file pcbi.1001017.s005.doc]

**Table S3. Prediction of large scale MADS interaction data**a

| **Species** | **Precision** | **Recall** | **F-score** | **Seqid-based**  **F-score** |
| --- | --- | --- | --- | --- |
| Tomato | 0.59 | 0.43 | 0.50 | 0.72 |
| Gerbera | 0.34 | 0.85 | 0.49 | 0.50 |

^a^ Performance for MADS interaction datasets for tomato [1] and gerbera [2].

**References**

*1. Leseberg CH, Eissler CL, Wang X, Johns MA, Duvall MR, et al. (2008) Interaction study of MADS-domain proteins in tomato. Journal of Experimental Botany 59: 2253-2265.*

*2. Ruokolainen S, Ng YP, Albert V, Elomaa P, Teeri T (2010) Large scale interaction analysis predicts that the Gerbera hybrida floral E function is provided both by general and specialized proteins. BMC Plant Biology 10: 129.*
