# Supplementary material for: Sequence Motifs in MADS Transcription Factors Responsible for Specificity and Diversification of Protein-Protein Interaction
Source: PLoS Comput Biol. 2010 Nov 24;6(11):e1001017. doi: 10.1371/journal.pcbi.1001017 (PMC2991254; doi:10.1371/journal.pcbi.1001017)
Supplement: Table S4 — Interaction prediction for SEP-homologs. (0.05 MB DOC) [file pcbi.1001017.s006.doc]

Table S4. Interaction prediction for SEP-homologs

| **Species/Interactions^a^** | **Experimental interaction^b^** | **Sequence similarity prediction^b^** | **IMSS based prediction^b^** |
| --- | --- | --- | --- |
| **Gerbera** |  |  |  |
| EST17F05-*GRCD5CTD* | y | n | y |
| EST21A03-*GRCD5CTD* | y | n | y |
| *GRCD1-GRCD4CTD* | y | n | y |
| *GRCD2-GRCD4CTD* | y | n | y |
| *GRCD4CTD-GRCD4CTD* | y | n | y |
| *GRCD4CTD-GRCD5CTD* | y | n | y |
| *GRCD5CTD-GRCD5CTD* | y | n | y |
| **Tomato** |  |  |  |
| *LeMADS1-RIN* | y | n | y |
| *RIN-TM29* | y | n | y |
| *TM29***-**TM4 | n | y | n |
| **Petunia** |  |  |  |
| *FBP2***-**FBP22 | y | n | y |
| pMADS3-*FBP4* | n | y | n |
| *FBP23***-**FBP22 | n | y | n |
| *FBP23***-**FBP13 | n | y | n |
| FBP13-*FBP9* | n | y | n |
| FBP13-*FBP4* | n | y | n |
| *FBP4***-**pMADS4 | n | y | n |

^a^ Pairs of proteins for which at least one member of the pair is most similar to *Arabidopsis* SEP1 or SEP3 (in italics), and for which (non-)interaction is not correctly predicted based on sequence similarity but correctly predicted based on IMSS interaction motifs.

^b^ “y” indicates interaction (experimental or predicted) and “n” indicates no interaction.
