# Supplementary material for: Sequence Motifs in MADS Transcription Factors Responsible for Specificity and Diversification of Protein-Protein Interaction
Source: PLoS Comput Biol. 2010 Nov 24;6(11):e1001017. doi: 10.1371/journal.pcbi.1001017 (PMC2991254; doi:10.1371/journal.pcbi.1001017)
Supplement: Table S5 — Mutagenesis positions. (0.06 MB DOC) [file pcbi.1001017.s007.doc]

Table S5. Mutagenesis positions

| **Protein** | **Mutation** | **“Ara_orig” ^a^** | **“Ara_new” ^a^** | **“All_species” ^a^** | **F-pred^b^** | **F-null^b^** |
| --- | --- | --- | --- | --- | --- | --- |
| SVP1 | C58S |  | x | x | 0.72 | 0.87 |
| SVP1 | S61R |  | x | x | 0.35 | 0.48 |
| SVP1 | C58S / S61R |  | x | x | 0.40 | 0.37 |
| SVP1 | EFCSSS56-61D |  | x | x | 0.50 | 0.17 |
| AGL24 | R61S | x | x | x | 0.78 | 0.73 |
| AGL14 | SIPK62-65MQD | x | x | x | 0.71 | 0.56 |
| SOC1 | MQD62-64SIPK | x | x | x | 0.63 | 0.38 |
| AP1 | I66V | x |  |  | 0.67 | 0.47 |
| CAL | V66I | x |  |  | 0.11 | 0.11 |
| AP1 | I66V / Y148N | x |  |  | 0.0 | 0.15 |
| CAL | V66I / N150Y | x |  |  | 0.0 | 0.0 |
| AG | Q126H | x |  |  | 0.50 | 0.53 |
| AP1 | Y148N | x |  |  | 0.67 | 0.61 |
| CAL | N150Y | x |  |  | 0.11 | 0.11 |
| SVP1 | SS227-228MF |  | x | x | 0.65 | 0.86 |

**^a^** For the three different IMSS models (for details see main text), ‘x’ indicates that the mutation changes the motif composition of the mutated protein.

**^b^** F-scores for prediction according to “ara_new” model (F-pred) and for a null model that considers the original interaction pattern as prediction of the interactions for the mutated protein (F-null).
