# Supplementary material for: Sequence Motifs in MADS Transcription Factors Responsible for Specificity and Diversification of Protein-Protein Interaction
Source: PLoS Comput Biol. 2010 Nov 24;6(11):e1001017. doi: 10.1371/journal.pcbi.1001017 (PMC2991254; doi:10.1371/journal.pcbi.1001017)
Supplement: Table S6 — Results yeast two-hybrid assays. (0.48 MB DOC) [file pcbi.1001017.s008.doc]

Table S6. Results yeast two-hybrid assays

| **MADS protein combinations^a^** | **- LWH + 1mM 3AT(1)** | **- LWH + 5mM 3AT (1)** | **- LWA (1)** | **- LWH + 1mM 3AT (2)** | **- LWH + 5mM 3AT (2)** | **- LWA (2)** | **Interac-tors** |
| --- | --- | --- | --- | --- | --- | --- | --- |
| **SVP1 C58S** |  |  |  |  |  |  |  |
| AD-SVP1 C58S x BD-AG | + | + | + | + | + | + | **AG** |
| AD-SVP1 C58S x BD-SHP1 | + | +/- | + | + | - | + | **SHP1** |
| BD-SVP1 C58S x AD-SEP1 | + | + | + | + | + | + | **SEP1** |
| AD-SVP1 C58S x BD-SEP4II | + | + | + | + | + | +/- | **SEP4II** |
| AD-SVP1 C58S x BD-SEP2 | + | + | + | + | + | +/- | **SEP2** |
| AD-SVP1 C58S x BD-SHP2 | + | +/- | + | + | - | +/- | **SHP2** |
| AD-SVP1 C58S x BD-AP1 | + | + | + | + | + | + |  |
| BD-SVP1 C58S x AD-AP1 | + | - | - | +/- | +/- | - | **AP1** |
| AD-SVP1 C58S x BD-FUL | + | + | + | + | + | + | **FUL** |
| AD-SVP1 C58S x BD-SEP3dC | + | + | + | + | + | + |  |
| BD-SVP1 C58S x AD-SEP3 | + | + | + | + | + | +/- | **SEP3** |
| AD-SVP1 C58S x BD-AGL14 | + | + | +/- | + | +/- | - | **AGL14** |
| AD-SVP1 C58S x BD-AGL16 | + | + | + | + | + | +/- | **AGL16** |
| AD-SVP1 C58S x BD-AGL17 | + | + | +/- | + | +/- | - | **AGL17** |
| AD-SVP1 C58S x BD-AGL19 | - | - | - | + | - | - |  |
| AD-SVP1 C58S x BD-SOC1 | + | + | + | + | + | + |  |
| BD-SVP1 C58S x AD-SOC1 | + | + | + | + | + | + | **SOC1** |
| AD-SVP1 C58S x BD-AGL21 | + | + | + | + | + | + |  |
| BD-SVP1 C58S x AD-AGL21 | + | + | + | + | + | + | **AGL21** |
| AD-SVP1 C58S x BD-SVP1 | + | + | - | + | - | - |  |
| BD-SVP1 C58S x AD-SVP1 | + | +/- | - | +/- | - | - |  |
| AD-SVP1 C58S x BD-SVP2 | + | + | + | + | + | +/- | **SVP2** |
| AD-SVP1 C58S x BD-FLC | + | +/- | - | + | + | + | **FLC** |
| AD-SVP1 C58S x BD-MAF2 | + | + | - | + | + | - |  |
| AD-SVP1 C58S x BD-AGL42 | + | + | + | + | +/- | + |  |
| BD-SVP1 C58S x AD-AGL42 | + | +/- | +/- | + | + | + | **AGL42** |
| AD-SVP1 C58S x BD-ANR1 | + | +/- | - | +/- | - | - |  |
| **SVP1 S61R** |  |  |  |  |  |  |  |
| AD-SVP1 S61R x BD-AG | +/- | - | - | + | + | + | **AG** |
| BD-SVP1 S61R x AD-SEP1 | + | + | + | + | + | + | **SEP1** |
| AD-SVP1 S61R x BD-SEP2 | +/- | - | - | + | + | + | **SEP2** |
| AD-SVP1 S61R x BD-SEP3dC | + | + | - | + | + | + |  |
| BD-SVP1 S61R x AD-SEP3 | + | + | + | + | + | + | **SEP3** |
| AD-SVP1 S61R x BD-AP1 | + | + | + | + | + | + |  |
| BD-SVP1 S61R x AD-AP1 | + | + | - | + | +/- | - | **AP1** |
| AD-SVP1 S61R x BD-AGL13 | + | + | +/- | - | - | - |  |
| AD-SVP1 S61R x BD-AGL16 | +/- | - | - | + | + | - |  |
| AD-SVP1 S61R x BD-SOC1 | + | - | - | + | + | - |  |
| BD-SVP1 S61R x AD-SOC1 | + | + | + | + | + | + | **SOC1** |
| AD-SVP1 S61R x BD-AGL21 | +/- | - | - | - | - | - |  |
| BD-SVP1 S61R x AD-AGL21 | + | + | + | + | + | + | **AGL21** |
| **SVP1 C58S/S61R** |  |  |  |  |  |  |  |
| AD-SVP1 C58S/S61R x BD-SHP1 | + | - | - | - | - | - |  |
| AD-SVP1 C58S/S61R x BD-AP1 | - | - | - | + | +/- | - |  |
| AD-SVP1 C58S/S61R x BD-SEP3dC | + | + | +/- | + | + | - | **SEP3** |
| BD-SVP1 C58S/S61R x AD-SEP3dC | + | +/- | - | + | - | - |  |
| AD-SVP1 C58S/S61R x BD-AGL16 | + | +/- | +/- | + | +/- | - | **AGL16** |
| AD-SVP1 C58S/S61R x BD-AGL19 | + | +/- | +/- | + | +/- | - | **AGL19** |
| AD-SVP1 C58S/S61R x BD-SOC1 | + | + | - | + | +/- | - |  |
| BD-SVP1 C58S/S61R x AD-SOC1 | + | + | + | + | + | + | **SOC1** |
| BD-SVP1 C58S/S61R x AD-AGL21 | + | + | + | + | + | + | **AGL21** |
| **SVP1 EFCSSS56-61D** |  |  |  |  |  |  |  |
| AD-SVP1 EFCSSS56-61D x BD-AGL19 | +/- | - | - | + | - | - |  |
| BD-SVP1 EFCSSS56-61D x AD-SOC1 | +/- | - | +/- | + | +/- | - | **SOC1** |
| BD-SVP1 EFCSSS56-61D x AD-AGL21 | +/- | - | +/- | + | - | +/- | **AGL21** |
| **SVP1 SS227-228MF** |  |  |  |  |  |  |  |
| AD-SVP1 SS227-228MF x BD-AG | + | + | + | + | + | + | **AG** |
| AD-SVP1 SS227-228MF x BD-SHP1 | + | + | + | + | + | + | **SHP1** |
| BD-SVP1 SS227-228MF x AD-SEP1 | + | + | + | + | + | + | **SEP1** |
| AD-SVP1 SS227-228MF x BD-SEP4I | + | + | + |  |  |  | **SEP4I** |
| AD-SVP1 SS227-228MF x BD-SEP4II | + | + | + | + | + | + | **SEP4II** |
| AD-SVP1 SS227-228MF x BD-SEP2 | + | + | + | + | + | + | **SEP2** |
| AD-SVP1 SS227-228MF x BD-SHP2 | + | + | + | + | + | + | **SHP2** |
| AD-SVP1 SS227-228MF x BD-AP1 | + | + | + | + | + | + |  |
| BD-SVP1 SS227-228MF x AD-AP1 | + | + | - | + | + | - | **AP1** |
| AD-SVP1 SS227-228MF x BD-FUL | + | + | +/- | + | + | + | **FUL** |
| AD-SVP1 SS227-228MF x BD-SEP3dC | + | + | + | + | + | + |  |
| BD-SVP1 SS227-228MF x AD-SEP3 | + | + | + | + | + | + | **SEP3** |
| AD-SVP1 SS227-228MF x BD-CAL | + | +/- | - | + | - | +/- | **CAL** |
| AD-SVP1 SS227-228MF x BD-STK | +/- | - | - | - | - | - |  |
| AD-SVP1 SS227-228MF x BD-AGL13 | + | +/- | - | + | - | +/- | **AGL13** |
| AD-SVP1 SS227-228MF x BD-AGL14 | + | + | + | + | + | + |  |
| BD-SVP1 SS227-228MF x AD-AGL14 | + | +/- | - | + | + | - | **AGL14** |
| BD-SVP1 SS227-228MF x AD-AGL15 | + | - | - | + | + | - |  |
| AD-SVP1 SS227-228MF x BD-AGL16 | + | + | + | + | + | + |  |
| BD-SVP1 SS227-228MF x AD-AGL16 | + | - | - | + | - | +/- | **AGL16** |
| AD-SVP1 SS227-228MF x BD-AGL17 | + | + | + | + | + | + | **AGL17** |
| AD-SVP1 SS227-228MF x BD-AGL19 | - | - | - | + | - | - |  |
| AD-SVP1 SS227-228MF x BD-SOC1 | + | + | + | + | + | + |  |
| BD-SVP1 SS227-228MF x AD-SOC1 | + | + | + | + | + | + | **SOC1** |
| AD-SVP1 SS227-228MF x BD-AGL21 | + | + | + | + | - | + |  |
| BD-SVP1 SS227-228MF x AD-AGL21 | + | + | + | + | + | + | **AGL21** |
| AD-SVP1 SS227-228MF x BD-SVP1 | + | + | - | + | + | - |  |
| BD-SVP1 SS227-228MF x AD-SVP1 | + | + | - | + | + | - |  |
| AD-SVP1 SS227-228MF x BD-SVP2 | + | + | + | + | - | + | **SVP2** |
| AD-SVP1 SS227-228MF x BD-FLC | + | + | +/- | + | + | + | **FLC** |
| BD-SVP1 SS227-228MF x AD-MAF2 | + | + | - | + | + | - |  |
| AD-SVP1 SS227-228MF x BD-MAF2 | + | + | + | - | - | - | **MAF2** |
| BD-SVP1 SS227-228MF x AD-ABSI | - | - | - | + | +/- | - |  |
| BD-SVP1 SS227-228MF x AD-ABSII | - | - | - | + | +/- | - |  |
| AD-SVP1 SS227-228MF x BD-AGL42 | + | + | + | + | + | + |  |
| BD-SVP1 SS227-228MF x AD-AGL42 |  |  |  | + | + | + | **AGL42** |
| AD-SVP1 SS227-228MF x BD-ANR1 | + | + | +/- | + | + | + | **ANR1** |
| **AGL24 R61S** |  |  |  |  |  |  |  |
| AD-AGL24 R61S x BD-AG | + | + | + | + | + | + | **AG** |
| BD-AGL24 R61S x AD-SEP1 | + | + | + | + | + | + | **SEP1** |
| AD-AGL24 R61S x BD-SHP1 | + | + | + | + | + | + | **SHP1** |
| AD-AGL24 R61S x BD-SEP4II | + | + | + | + | + | + | **SEP4II** |
| AD-AGL24 R61S x BD-SEP2 | + | + | + | + | + | + | **SEP2** |
| AD-AGL24 R61S x BD-SHP2 | + | + | + | + | + | + | **SHP2** |
| AD-AGL24 R61S x BD-SEP3dC | + | + | + | + | + | + |  |
| BD-AGL24 R61S x AD-SEP3 | + | + | + | + | + | + | **SEP3** |
| AD-AGL24 R61S x BD-AP1 | + | + | + | + | + | + |  |
| BD-AGL24 R61S x AD-AP1 | + | +/- | - | + | + | - | **AP1** |
| AD-AGL24 R61S x BD-FUL | + | + | + | + | + | + |  |
| BD-AGL24 R61S x AD-FUL | + | + | - | + | + | - | **FUL** |
| AD-AGL24 R61S x BD-STK | + | +/- | + | + | - | +/- | **STK** |
| AD-AGL24 R61S x BD-AGL12 | - | - | - | + | - | - |  |
| AD-AGL24 R61S x BD-AGL14 | + | + | + | + | + | + |  |
| BD-AGL24 R61S x AD-AGL14 | + | + | + | + | + | + | **AGL14** |
| BD-AGL24 R61S x AD-AGL15 | + | - | - | + | - | - |  |
| AD-AGL24 R61S x BD-AGL16 | + | + | + | + | + | + | **AGL16** |
| AD-AGL24 R61S x BD-AGL19 | +/- | - | - | +/- | - | - |  |
| BD-AGL24 R61S x AD-AGL19 | - | - | - | + | +/- | - |  |
| AD-AGL24 R61S x BD-SOC1 | + | + | + | + | + | + |  |
| BD-AGL24 R61S x AD-SOC1 | + | + | + | + | + | + | **SOC1** |
| AD-AGL24 R61S x BD-AGL21 | +/- | - | +/- | + | - | - |  |
| BD-AGL24 R61S x AD-AGL21 | + | - | + | + | +/- | + | **AGL21** |
| AD-AGL24 R61S x BD-AGL24 | + | + | + | + | + | + |  |
| BD-AGL24 R61S x AD-AGL24 | + | + | + | + | + | + | **AGL24** |
| BD-AGL24 R61S x AD-MAF2 | + | +/- | +/- | + | + | + |  |
| AD-AGL24 R61S x BD-MAF2 | + | + | + | + | + | + | **MAF2** |
| BD-AGL24 R61S x AD-ABSI | - | - | - | + | - | + |  |
| AD-AGL24 R61S x BD-AGL42 | + | + | + | + | + | + |  |
| BD-AGL24 R61S x AD-AGL42 | + | +/- | +/- | + | +/- | + | **AGL42** |
| **AGL14 SIPK62-65MQD** |  |  |  |  |  |  |  |
| AD-AGL14 SIPK62-65MQD x BD-AG | + | - | - | + | - | - |  |
| AD-AGL14 SIPK62-65MQD x BD-SEP2 | + | +/- | + | + | +/- | + | **SEP2** |
| AD-AGL14 SIPK62-65MQD x BD-SHP2 | + | + | +/- | + | + | +/- | **SHP2** |
| AD-AGL14 SIPK62-65MQD x BD-SEP3dC | + | + | + | + | + | + | **SEP3** |
| AD-AGL14 SIPK62-65MQD x BD-AP1 | + | + | + | + | + | + | **AP1** |
| AD-AGL14 SIPK62-65MQD x BD-FUL | + | + | + | + | + | + | **FUL** |
| AD-AGL14 SIPK62-65MQD x BD-AGL12 | + | - | +/- | + | +/- | +/- | **AGL12** |
| AD-AGL14 SIPK62-65MQD x BD-AGL13 | + | + | + | + | + | + | **AGL13** |
| AD-AGL14 SIPK62-65MQD x BD-AGL14 | + | + | - | + | + | - |  |
| AD-AGL14 SIPK62-65MQD x BD-AGL16 | + | + | + | + | + | + | **AGL16** |
| AD-AGL14 SIPK62-65MQD x BD-SOC1 | + | + | + | + | + | + | **SOC1** |
| AD-AGL14 SIPK62-65MQD x BD-AGL21 | + | - | - | + | - | - |  |
| AD-AGL14 SIPK62-65MQD x BD-SVP1 | + | + | + | + | + | + | **SVP1** |
| AD-AGL14 SIPK62-65MQD x BD-SVP2 | + | + | + | + | + | + | **SVP2** |
| AD-AGL14 SIPK62-65MQD x BD-AGL24 | + | + | + | + | + | + | **AGL24** |
| AD-AGL14 SIPK62-65MQD x BD-ANR1 | + | - | +/- | + | - | +/- | **ANR1** |
| **SOC1 MQD62-64SIPK** |  |  |  |  |  |  |  |
| BD-SOC1 MQD62-64SIPK x AD-SEP1 | + | + | + | + | + | + | **SEP1** |
| BD-SOC1 MQD62-64SIPK x AD-SEP3 | + | + | + | + | + | + | **SEP3** |
| BD-SOC1 MQD62-64SIPK x AD-AGL13 | + | +/- | - | + | +/- | - |  |
| BD-SOC1 MQD62-64SIPK x AD-SOC1 | + | + | +/- | + | +/- | +/- | **SOC1** |
| BD-SOC1 MQD62-64SIPK x AD-AGL21 | + | + | + | + | + | + | **AGL21** |
| BD-SOC1 MQD62-64SIPK x AD-SVP1 | + | + | + | + | + | + | **SVP1** |
| BD-SOC1 MQD62-64SIPK x AD-AGL24 | + | + | + | + | + | + | **AGL24** |
| **AP1 I66V** |  |  |  |  |  |  |  |
| BD-AP1 I66V x AD-SEP1 | + | +/- | +/- | + | +/- | +/- | **SEP1** |
| AD-AP1 I66V x BD-SEP2 | + | - | + | + | - | + | **SEP2** |
| AD-AP1 I66V x BD-SEP3dC | + | + | + | + | + | + |  |
| BD-AP1 I66V x AD-SEP3 | + | +/- | - | + | - | - | **SEP3** |
| AD-AP1 I66V x BD-AGL16 | + | + | + | + | + | + | **AGL16** |
| AD-AP1 I66V x BD-AGL19 | +/- | - | - | +/- | - | - |  |
| AD-AP1 I66V x BD-SOC1 | + | - | +/- | +/- | - | +/- | **SOC1** |
| **AP1 Y148N** |  |  |  |  |  |  |  |
| AD-AP1 Y148N x BD-AG | +/- | - | - | +/- | - | - |  |
| BD-AP1 Y148N x AD-SEP1 | + | +/- | +/- | + | +/- | +/- | **SEP1** |
| AD-AP1 Y148N x BD-SEP2 | + | - | + | + | - | + | **SEP2** |
| AD-AP1 Y148N x BD-SEP3dC | + | + | + | + | + | + |  |
| BD-AP1 Y148N x AD-SEP3 | + | + | +/- | + | + | +/- | **SEP3** |
| AD-AP1 Y148N x BD-AP1 | + | + | + | + | + | + | **AP1** |
| AD-AP1 Y148N x BD-FUL | + | + | + | + | + | + | **FUL** |
| AD-AP1 Y148N x BD-AGL13 | + | +/- | +/- | + | +/- | +/- | **AGL13** |
| AD-AP1 Y148N x BD-AGL16 | + | + | + | + | + | + | **AGL16** |
| AD-AP1 Y148N x BD-AGL19 | +/- | - | - | +/- | - | - |  |
| AD-AP1 Y148N x BD-SOC1 | + | + | + | + | + | + | **SOC1** |
| AD-AP1 Y148N x BD-AGL21 | +/- | - | - | +/- | - | - |  |
| AD-AP1 Y148N x BD-SVP1 | + | + | +/- | + | + | +/- | **SVP1** |
| AD-AP1 Y148N x BD-SVP2 | + | + | + | + | + | + | **SVP2** |
| AD-AP1 Y148N x BD-AGL24 | + | + | + | + | + | + | **AGL24** |
| **AP1 I66V/Y148N** |  |  |  |  |  |  |  |
| AD-AP1 I66V/Y148N x BD-SEP4II | + | +/- | +/- | + | +/- | +/- | **SEP4II** |
| **CAL V66I** |  |  |  |  |  |  |  |
| BD CAL V66I x AD-SEP1 | + | + | + | + | + | + | **SEP1** |
| AD CAL V66I x BD-SEP2 | + | +/- | + | + | +/- | + | **SEP2** |
| AD CAL V66I x BD-SHP2 | + | + | +/- | + | + | +/- | **SHP2** |
| AD CAL V66I x BD-SEP3dC | + | + | + | + | + | + |  |
| BD CAL V66I x AD-SEP3 | + | - | +/- | + | +/- | +/- | **SEP3** |
| AD CAL V66I x BD-AP1 | + | + | + | + | + | + | **AP1** |
| AD CAL V66I x BD-FUL | + | + | + | + | + | + | **FUL** |
| AD CAL V66I x BD-AGL12 | + | - | +/- | + | +/- | +/- | **AGL12** |
| AD CAL V66I x BD-AGL13 | + | + | + | + | + | + | **AGL13** |
| AD CAL V66I x BD-AGL14 | + | +/- | - | + | +/- | - |  |
| AD CAL V66I x BD-AGL16 | + | + | + | + | + | + | **AGL16** |
| AD CAL V66I x BD-SOC1 | + | + | + | + | + | + | **SOC1** |
| AD CAL V66I x BD-AGL21 | +/- | - | - | +/- | - | - |  |
| AD CAL V66I x BD-SVP1 | + | + | + | + | + | + | **SVP1** |
| AD CAL V66I x BD-SVP2 | + | + | + | + | + | + | **SVP2** |
| AD CAL V66I x BD-AGL24 | + | + | + | + | + | + | **AGL24** |
| AD CAL V66I x BD-ANR1 | + | +/- | - | + | + | - |  |
| **CAL N150Y** |  |  |  |  |  |  |  |
| AD-CAL N150Y x BD-AG | + | + | + | + | + | + | **AG** |
| AD-CAL N150Y x BD-SHP1 | + | + | + | + | + | + | **SHP1** |
| BD-CAL N150Y x AD-SEP1 | + | + | + | + | + | + | **SEP1** |
| AD-CAL N150Y x BD-SEP4II | + | + | + | + | + | + | **SEP4II** |
| AD-CAL N150Y x BD-SEP2 | + | + | + | + | + | + | **SEP2** |
| AD-CAL N150Y x BD-SHP2 | + | + | + | + | + | + | **SHP2** |
| AD-CAL N150Y x BD-SEP3dC | + | + | + | + | + | + | **SEP3** |
| AD-CAL N150Y x BD-AP1 | + | + | + | + | + | + | **AP1** |
| AD-CAL N150Y x BD-FUL | + | + | + | + | + | + | **FUL** |
| AD-CAL N150Y x BD-AGL13 | +/- | - | +/- | +/- | +/- | +/- | **AGL13** |
| AD-CAL N150Y x BD-AGL14 | + | + | + | + | + | + | **AGL14** |
| AD-CAL N150Y x BD-AGL16 | + | - | +/- | + | - | +/- | **AGL16** |
| AD-CAL N150Y x BD-AGL19 | +/- | - | - | +/- | - | - |  |
| AD-CAL N150Y x BD-SOC1 | + | + | + | + | + | + | **SOC1** |
| AD-CAL N150Y x BD-AGL21 | +/- | - | - | +/- | - | - |  |
| AD-CAL N150Y x BD-SVP1 | + | + | + | + | + | + | **SVP1** |
| AD-CAL N150Y x BD-SVP2 | + | + | + | + | + | + | **SVP2** |
| AD-CAL N150Y x BD-AGL24 | + | + | + | + | + | + | **AGL24** |
| AD-CAL N150Y x BD-AGL42 | + | + | + | + | + | + | **AGL42** |
| AD-CAL N150Y x BD-ANR1 | + | - | - | + | +/- | - |  |
| **CAL V66I/N150Y** |  |  |  |  |  |  | **none** |
| **AG Q126H** |  |  |  |  |  |  |  |
| BD-AG Q126H x AD-SEP1 | + | + | + | + | + | + | **SEP1** |
| AD-AG Q126H x BD-SEP2 | + | +/- | + | + | - | + | **SEP2** |
| AD-AG Q126H x BD-SEP3dC | + | + | + | + | + | + | **SEP3** |
| AD-AG Q126H x BD-AP1 | - | - | - | - | - | +/- |  |
| AD-AG Q126H x BD-AGL16 | + | + | + | + | + | + | **AGL16** |

^a^All possible combinations have been tested reciprocally in a matrix-based yeast two-hybrid assay in duplicate. Only combinations that once gave growth of yeast are indicated in the table. In the last column MADS domain proteins are listed that are regarded as interaction partners for the individual mutated proteins, based on our selection criteria (see Methods section in main text). CAL V661/N150Y is the only protein tested that was not interacting with any other MADS domain protein. “-“, no yeast growth; “+/-“, moderate yeast growth; “+” substantial yeast growth.
