# Supplementary material for: Sequence Motifs in MADS Transcription Factors Responsible for Specificity and Diversification of Protein-Protein Interaction
Source: PLoS Comput Biol. 2010 Nov 24;6(11):e1001017. doi: 10.1371/journal.pcbi.1001017 (PMC2991254; doi:10.1371/journal.pcbi.1001017)
Supplement: Table S7 — Analysis of known mutations in MADS domain proteins. (0.08 MB DOC) [file pcbi.1001017.s009.doc]

**Table S7. Analysis of known mutations in MADS proteins**

| **MADS** | **Residues** | **Overlap with IMSS** | **Reference^a^** |
| --- | --- | --- | --- |
| AGL15-AGL15 | 118 - 152 | 118-123 | [1] |
| OsMADS1, OsMADS14, OsMADS15 | K-domain and region before K | N/A | [2] |
| OsMADS1 | Arg24 | N/A | [3] |
| SOC1 | Glu34, Gly113 | - | [4] |
| SEP1 (interaction with PI) | K143 | - | [5] |
| PI (interaction with SEP1) | E125 | - | [5] |
| SEP3 (interaction with PI) | R146 | - | [5] |
| AP1 – AG | MADS and I domain | agrees with motifs | [6] |
| AP3 – PI | I and K domain | no motifs for AP3 | [6] |
| ABS (interaction with SEP3) | Distal part of K-domain | no motifs for ABS | [7] |
| AP3-PI | Proximal part of K-domain | PI: 100-107, 109-116 | [8] |
| OsMADS6 | 109-137 | N/A | [9] |
| Antirrhinum DEF - GLO | K93 deletion, F49 insertion | N/A | [10] |
| SQUA-SQUA | MADS/I only cannot dimerize with MADS/I/K | N/A |  |
| SQUA-PLE | MADS/I can dimerize with MADS/I/K | N/A | [11] |
| AP1 / CAL | Distinct residues of AP1 needed for recovery of floral meristem identity | AP1: 151-158 | [12] |
| CAL | Cal-4: E131K | 126-133 | [13] |

**^a^ References**

*1. Hill K, Wang H, Perry SE (2008) A transcriptional repression motif in the MADS factor AGL15 is involved in recruitment of histone deacetylase complex components. Plant Journal 53: 172-185.*

*2. Lim J, Moon YH, An G, Jang SK (2000) Two rice MADS domain proteins interact with OsMADS1. Plant Molecular Biology 44: 513-527.*

*3. Jeon JS, Jang S, Lee S, Nam J, Kim C, et al. (2000) leafy hull sterile1 is a homeotic mutation in a rice MADS box gene affecting rice flower development. Plant Cell 12: 871-884.*

*4. Lee J, Oh M, Park H, Lee I (2008) SOC1 translocated to the nucleus by interaction with AGL24 directly regulates LEAFY. Plant Journal 55: 832-843.*

*5. Yang YZ, Jack T (2004) Defining subdomains of the K domain important for protein-protein interactions of plant MADS proteins. Plant Molecular Biology 55: 45-59.*

*6. Krizek BA, Meyerowitz EM (1996) Mapping the protein regions responsible for the functional specificities of the Arabidopsis MADS domain organ-identity proteins. Proceedings of the National Academy of Sciences of the United States of America 93: 4063-4070.*

*7. Kaufmann K, Anfang N, Saedler H, Theissen G (2005) Mutant analysis, protein-protein interactions and subcellular localization of the Arabidopsis B-sister (ABS) protein. Molecular Genetics and Genomics 274: 103-118.*

*8. Yang YZ, Fanning L, Jack T (2003) The K domain mediates heterodimerization of the Arabidopsis floral organ identity proteins, APETALA3 and PISTILLATA. Plant Journal 33: 47-59.*

*9. Moon YH, Kang HG, Jung JY, Jeon JS, Sung SK, et al. (1999) Determination of the motif responsible for interaction between the rice APETALA1/AGAMOUS-LIKE9 family proteins using a yeast two-hybrid system. Plant Physiology 120: 1193-1203.*

*10. Zachgo S, Silva ED, Motte P, Trobner W, Saedler H, et al. (1995) Functional-Analysis of the Antirrhinum Floral Homeotic Deficiens Gene in-Vivo and in-Vitro by Using a Temperature-Sensitive Mutant. Development 121: 2861-2875.*

*11. West AG, Causier BE, Davies B, Sharrocks AD (1998) DNA binding and dimerisation determinants of Antirrhinum majus MADS-box transcription factors. Nucleic Acids Research 26: 5277-5287.*

*12. Alvarez-Buylla ER, Garcia-Ponce B, Garay-Arroyo A (2006) Unique and redundant functional domains of APETALA1 and CAULIFLOWER, two recently duplicated Arabidopsis thaliana floral MADS-box genes. Journal of Experimental Botany 57: 3099-3107.*

*13. Kempin SA, Savidge B, Yanofsky MF (1995) Molecular-Basis of the Cauliflower Phenotype in Arabidopsis. Science 267: 522-525.*
