# Supplementary material for: Sequence Motifs in MADS Transcription Factors Responsible for Specificity and Diversification of Protein-Protein Interaction
Source: PLoS Comput Biol. 2010 Nov 24;6(11):e1001017. doi: 10.1371/journal.pcbi.1001017 (PMC2991254; doi:10.1371/journal.pcbi.1001017)
Supplement: Table S8 — Previous computational studies that revealed residues that are under functional constraint. (0.05 MB DOC) [file pcbi.1001017.s010.doc]

**Table S8. Previous computational studies that revealed residues that are under functional constraint**

| **MADS** | **Residues** | **Computational approach** | **Reference^a^** |
| --- | --- | --- | --- |
| FLC | 42, 56, 59, 154 | Analysis of positive selection | [1] |
| SVP | 16, 144 | Analysis of positive selection | [1] |
| Petunia MADS | Regions in several proteins | Window analysis to identify regions with rate differences | [2] |
| PI | 100N, 142A | Analysis of substitution rates | [3] |
| AP3 | 148R, 162C, 130E, 191K | Analysis of substitution rates | [3] |

^a^References:

*1. Martinez-Castilla LP, Alvarez-Buylla ER (2003) Adaptive evolution in the Arabidopsis MADS-box gene family inferred from its complete resolved phylogeny. Proceedings of the National Academy of Sciences of the United States of America 100: 13407-13412.*

*2. Nam J, Kaufmann K, Theiben G, Nei M (2005) A simple method for predicting the functional differentiation of duplicate genes and its application to MIKC-type MADS-box genes. Nucleic Acids Research 33: -.*

*3. Hernandez-Hernandez T, Martinez-Castilla LP, Alvarez-Buylla ER (2007) Functional diversification of B MADS-Box homeotic regulators of flower development: Adaptive evolution in protein-protein interaction domains after major gene duplication events. Molecular Biology and Evolution 24: 465-481.*
