# Supplementary material for: Sequence Motifs in MADS Transcription Factors Responsible for Specificity and Diversification of Protein-Protein Interaction
Source: PLoS Comput Biol. 2010 Nov 24;6(11):e1001017. doi: 10.1371/journal.pcbi.1001017 (PMC2991254; doi:10.1371/journal.pcbi.1001017)
Supplement: Table S9 — SEP equivalents grouped according to expression in whorl one. (0.05 MB DOC) [file pcbi.1001017.s011.doc]

**Table S9. SEP equivalents grouped according to expression in whorl one**

^a^ Protein name and motif in italics indicates motif present

| **Expression in whorl 1:**  **Only 2 out of 9 contain motif^a^** | |
| --- | --- |
| AdOM1 | SSTSMLKT |
| AmDEFH200 | SSTSMLNT |
| AmDEFH72 | NSGTMLKT |
| AoAM4 | SSPSMLKT |
| *AoAOM1* | *SSSSMLKT* |
| DgMADS1 | SSRSMLKT |
| *GhGRCD1* | *STSSMLKT* |
| SaMADSD | SSSSMIRT |
| SiSEP3 | SSPSMLKT |
| **No expression in whorl 1: 7 out of 11 contain motif^a^** | |
| *AtSEP3* | *SSSSMLRT* |
| BpMADS1 | SSPSMLKT |
| *EbAGL9* | *SSSSSMFK* |
| HvM9 | STQSMTKT |
| *LeTM5* | *SSSSMLKT* |
| *MgAGL9* | *SGSSMLKT* |
| *NsMADS3* | *SSSSMLKT* |
| OsMADS7 | STQSMTKT |
| OsMADS8 | SGQSMTRT |
| *PhFBP2* | *SSSSMLKT* |
| *PsMADS* | *STSSMLKT* |
