# Supplementary material for: Sequence Motifs in MADS Transcription Factors Responsible for Specificity and Diversification of Protein-Protein Interaction
Source: PLoS Comput Biol. 2010 Nov 24;6(11):e1001017. doi: 10.1371/journal.pcbi.1001017 (PMC2991254; doi:10.1371/journal.pcbi.1001017)
Supplement: Table S10 — SNPs overlapping IMSS motifs. (0.05 MB DOC) [file pcbi.1001017.s012.doc]

**Table S10. SNPs overlapping IMSS motifs**

| **MADS** | **Sequence** | **SNP** | **Ecotype in which SNP occurs** |
| --- | --- | --- | --- |
| AGL14 | ISSSS**S**TS | S->T | 62 out of 80 ecotypes |
| AGL14 | ISSSSS**T**S | T->S | 60 out of 80 ecotypes |
| AGL16 | MKSVI**E**RY | E->D | ice61, Istisu-1, Xan-1 |
| AGL16 | MKSVIER**Y** | Y->C | ice61 |
| AGL16 | SMKSVI**E**R | E->D | ice61 |
| AGL17 | FAS**S**SVKS | S->Y | Dog-4 |
| AGL18 | SMKGEL**E**R | E->K | Don-0 |
| AGL21 | GEQL**N**GLS | N->H | Bak-7 |
| AGL31 | MSKIID**R**Y | R->C | Kastel-1 |
| AGL42 | FSSSDM**Q**K | Q->R | Vie-0 |
| AGL42 | I**F**SQRGRL | F->S | ice7 |
| AGL42 | IF**S**QRGRL | S->P | ice7 |
| AGL42 | M**Q**KTIERY | Q->R | Vie-0 |
| AGL69 | YSFSS**G**DS | G->A | Don-0 |
| AGL71 | LEVHH**R**KL | R->G | ice49 |
| AGL71 | MEKII**D**RY | D->E | Cdm-0, Don-0 |
| AGL71 | **T**ELQEIDT | T->A | Cdm-0, Don-0 |
| AGL72 | QKGRLY**E**F | E->K | Cdm-0, Don-0 |
| AGL79 | M**E**RILDRY | E->G | ice79 |
| ANR1 | EEQH**Q**LLN | Q->E | ice111,ice169,ice173,ice181,ice21,ice36,ice63,  ice79,ice7,Istisu-1,Lerik1-3,Nie1-2,Tu-SB30-3,Tu-Scha-9,Tu-V-13,Xan-1,Fei-0 |
| CAL | HKGKLFE**Y** | Y->N | Don-0 |
| FUL | GEDL**D**SLS | D->E | Don-0, Ped-0 |
