# Supplementary material for: Sequence Motifs in MADS Transcription Factors Responsible for Specificity and Diversification of Protein-Protein Interaction
Source: PLoS Comput Biol. 2010 Nov 24;6(11):e1001017. doi: 10.1371/journal.pcbi.1001017 (PMC2991254; doi:10.1371/journal.pcbi.1001017)
Supplement: Table S11 — Pairs of sequences with indel overlapping predicted interaction motif. (0.07 MB DOC) [file pcbi.1001017.s013.doc]

**Table S11. Pairs of sequences with indel overlapping predicted interaction motif**

| **Species** | **Protein with deletion** | **Protein with insertion** | **Sequence around indel^a^** | **Pos. of indel** | **Clade** |
| --- | --- | --- | --- | --- | --- |
| **Both insertion and deletion occur within interaction motif** | | | | | |
| *Arabidopsis thaliana* | AGL42 | AGL14 | YEFsSS | 55 | TM3 |
| *Arabidopsis thaliana* | SEP1 | SEP4 | FCSSpS | 57 | AGL2 |
| *Arabidopsis thaliana* | AGL19 | AGL14 | YEFsSS | 55 | TM3 |
| *Antirrhinum majus* | DEFH49 | AmSEP3B | FCSSsS | 57 | AGL2 |
| *Brachypodium*  *distachyon* | Q0PLP4 | Q0PLP3 | SSSSfM | 183 | SQUA |
| *Ipomoea batatas* | Q9AT57 | Q9AT62 | ELQLvE | 85 | STMADS11 |
| *Magnolia*  *praecocissima* | Q948U6 | Q948U9 | QLERsL | 97 | TM3 |
| *Ophioglossum*  *pedunculosum* | Q70ZY4 | O24173 | SSMmRR | 23 | SQUA |
| *Oryza sativa* | Q8RU31 | Q40704 | QNANsR | 107 | AG |
| *Prunus dulcis* | Q6W3F2 | Q3KSZ0 | YEFCsS | 46 | AGL2 |
| *Prunus persica* | Q4F8B3 | A4GVG4 | YEFCsS | 55 | AGL2 |
| *Prunus persica* | A4GVG3 | A4GVG4 | YEFCsS | 55 | AGL2 |
| *Taihangia rupestris* | Q2EMR9 | Q2EMR8 | YEFCsS | 55 | AGL2 |
| *Triticum aestivum* | Q1G169 | Q1G184 | RVLqHP | 195 | AGL2 |
| *Zea mays* | ZMM1 | ZAG2 | PGSLqQ | 226 | AG |
| **Only protein with deletion contains motif; insertion disrupts motif** | | | | | |
| *Antirrhinum majus* | PLE | FAR | MQKRqE | 166 | AG |
| *Brassica napus* | Q1ZZ81 | Q39296 | NQEEdC | 87 | AGL15 |
| *Helleborus orientalis* | Q84Y45 | Q84Y46 | NGYhHQ | 153 | GLO |
| *Ipomoea nil* | Q76N61 | Q76N62 | QKRqEI | 171 | AG |
| *Ipomoea batatas* | Q9AT62 | Q9AT57 | SITtNV | 199 | STMADS11 |
| *Misopates*  *orontium* | Q2WBM3 | Q2WBM7 | MQKRqE | 164 | AG |
| *Petunia hybrida* | FBP9 | FBP23 | LNSSnS | 209 | AGL2 |
| **Only protein with insertion contains motif; deletion disrupts motif** | | | | | |
| *Gnetum gnemon* | Q9FST3 | Q9XGJ6 | KCSyAL | 71 | AGL2 |
| *Hordeum vulgare* | Q8RU43 | Q8RU44 | QNSNsR | 105 | AG |
| *Nicotiana tabacum* | Q8GT99 | Q0Q5E9 | GEAYqS | 210 | SQUA |
| *Oryza sativa* | Q0D4T4 | Q10CQ1 | VLQKeL | 167 | SQUA |
| *Taihangia rupestris* | Q2EMR9 | Q2EMR8 | SSSSsM | 59 | AGL2 |
| *Zea mays* | Q84V75 | O24009 | QNANtR | 137 | AG |
| *Zea mays* | Q9ZR64 | Q9M4P8 | DQLnNE | 202 | AGL2 |

^a^. Residues in lower case indicate position of indel.
