# Supplementary material for: Sequence Motifs in MADS Transcription Factors Responsible for Specificity and Diversification of Protein-Protein Interaction
Source: PLoS Comput Biol. 2010 Nov 24;6(11):e1001017. doi: 10.1371/journal.pcbi.1001017 (PMC2991254; doi:10.1371/journal.pcbi.1001017)
Supplement: Text S1 — Additional text van Dijk et al. (0.11 MB DOC) [file pcbi.1001017.s014.doc]

**Supporting text 1**

**Sequence motifs in MADS transcription factors responsible for specificity and diversification of protein-protein interactions.**

***Van Dijk et al.***

**Contents**

1. **Genome wide predictions for different plant species.**
2. **IMSS motif locations and mutagenesis positions.**
3. **Neo- or sub-functionalization deteriorates performance of sequence similarity based interaction prediction but not of motif based prediction.**
4. **Intron borders and alternative splicing.**
5. **Overlap between indels and interaction motifs.**

**1. Genome wide predictions for different plant species.** We applied our model on a genome-wide scale across plant species for which interaction data are available. Interactions as listed in the supplementary information of [1] were used. In addition, some newly acquired datasets were obtained [2-8] (Table S2). Note that we took care to only use interactions for which in the experiments the full-length MADS proteins have been used (in several cases in literature, the IKC region of MADS domain proteins has been used without the N-terminal DNA binding domain).

Note that after our model has been trained, in order to make new predictions for additional species, all that is needed are sequences of the relevant proteins. The method than first searches for the occurrence of interaction motifs in those sequences, and based on those it predicts interactions of those proteins. Scripts to perform this analysis can be obtained upon request.

The accuracy of these predictions, 60%, is somewhat lower than what is obtained based on simple sequence similarity (orthology-based predictions, 72% accuracy). For tomato, where in addition to information about protein-protein interactions also information about non-interactions are available, comparison between motif-based predictions and orthology-based predictions also shows that the latter performs somewhat better. For gerbera, however, the accuracy of both types of predictions is comparable (Table S3).

Moreover, there are several cases of interactions that are not correctly predicted based on overall sequence similarity but are correctly predicted based on the present interaction motifs, which points to cases of sub- or neo-functionalization. In particular, several of these involve pairs of proteins in which one protein of the pair is most similar to *Arabidopsis* SEPALLATA1 (SEP1) or SEP3 (see Text 3 below and Table S4).

**2. IMSS motif locations and mutagenesis positions.** Positions of IMSS motifs (based on “ara_new” model, see Main Text) in the various MADS domain proteins are listed in Table S1. Mutations were designed in a particular subset of MADS domain proteins taking the following three criteria into account: (1) Try to create gain of interaction partners by creating a putative interaction motif. (2) Determine the role and importance of motifs present at positions in the different conserved MADS transcription factor protein domains (MADS, I, K-box, C-region). (3) Try to swap interaction specificity between individual proteins by swapping a predicted interaction motif. All mutations were introduced as described in the Methods section and are listed in Table S5, where it is also indicated in which of the different models the mutations overlap with motifs. Below we explain for each individual protein or combination of proteins how the amino acid residues to be mutated were selected.

SVP1 C58S; SVP1 S61R; SVP1 C58S/S61R and AGL24 R61S

*AGAMOUS LIKE24 (AGL24)* and *SHORT VEGETATIVE PHASE (SVP)* are two closely related MADS box genes involved in regulation of floral timing [9,10]. Remarkably, *AGL24* acts as a dosage dependent activator of flowering, whereas *SVP* is a dosage dependent floral repressor. Alternative splicing occurs for the *SVP* gene, giving rise to at least three different mRNAs [11]. We named the protein encoded by the fully spliced version SVP1. Recently, it was shown that this protein dimerizes with the FLOWERING LOCUS C (FLC) protein, which is also known as a strong repressor of flowering [12]. In the large-scale yeast two-hybrid analysis performed by [1] a protein encoded by a second splice variant was used (named here SVP2). This protein has an altered C-terminal domain due to retention of the sixth intron in the messenger, which contains an in-frame stop codon [11]. Recently, a third splice variant was isolated from *Arabidopsis* inflorescences (GenBank accession EU078686), which encodes a protein lacking 5 amino acid residues at the ‘hotspot region’ located at the border between the MADS domain and the I-region and which overlaps with a predicted interaction motif (SVP3, see below). The goal of the first set of mutations was to swap the interaction specificity between SVP1 and AGL24. The proteins differ in their interaction pattern, which could be expected based on their opposite functions in floral timing, but also have a large overlap in interaction partners [1]; this study, Table 1). In line with the latter observation, various predicted interaction motifs are present in both SVP1 and AGL24 (Table S1). However, at the ‘hotspot region’ between the MADS domain and the I-region, SVP1 and AGL24 contain a different predicted interaction motif (F**C**SS**S**M**KE** and F**S**SS**R**M**RD**, respectively), which is therefore a good candidate position for determining the differences between these two proteins. The four amino acid residues that are different between AGL24 and SVP1 within this motif are indicated in bold in the motifs above. Comparison of putative SVP1 orthologs from various species showed that position two and five of the motif are very conserved, whereas positions seven and eight vary. The last position in fact does not seem to discriminate between AGL24 and SVP1-like proteins at all. Based on this knowledge we decided to swap the amino acids at position two (C/S) and five (S/R) of the motif between SVP1 and AGL24.

SVP1 EFCSSS56-61D (SVP3).

This mutated protein was generated because it reflects a natural occurring alternative splicing variant (GenBank accession EU078686) that lacks the interaction motif at the “hotspot region”. Based on the proposed importance of motifs at this position we expect that this relative small change in protein sequence will give a large decrease in protein-protein interaction capacity, as is predicted by IMSS.

SVP1 SS227-228MF

This mutation was generated in order to remove a motif present in the C-terminal region of SVP1, but not in the second splice variant named SVP2. The SS was changed into MF based on sequence alignment with SVP2. We expected to limit the number of SVP1 interaction partners by this mutation and to generate a protein that more resembles SVP2 in this respect.

AGL14 SIPK62-65MQD; SOC1 MQD62-64SIPK

The goal of this experiment was to swap interaction specificity of AGAMOUS LIKE14 (AGL14) and SUPRESSOR OF OVEREXPRESSION OF CO1 (SOC1). These two proteins are closely related [9], but differ substantially in their interaction capacity; whereas SOC1 is one of the MADS domain proteins with the most interaction partners, AGL14 has only a limited number of interactors [1]. Again, a pronounced difference was found at the “hotspot region”. 62-64MQD is part of three predicted overlapping interaction motifs that are not present in AGL14. Therefore, this position of the sequence was chosen to be swapped between AGL14 and SOC1. Besides this difference a few others are present so we expected a partial but substantial swap of interaction capacity.

AP1 I66V; AP1 Y148N; AP1 I66V/Y148N; CAL V66I; CAL N150Y; CAL V66I/N150Y

This set of single and double mutations were generated aiming to swap interaction specificity of these two closely related proteins that act partially redundant, but have a diverged interaction capacity [1,13]. Functional analyses revealed that APETALA1 (AP1) is able to fully substitute CAULIFLOWER (CAL) functions, but CAL can not take over all AP1 functions [14]. Subsequent domain swap experiments showed that the K-box, in which one of the predicted motifs that we mutated is contained, plays a pivotal role in the difference between AP1 and CAL. In contrast to AP1, the wild-type CAL protein has only a limited number of interaction partners [1]. Note that the motif in the K-box occurs only in the “ara_original” and not in the “ara_new” model. The other mutation targets a motif in the “hotspot” region (see Main Text). It is present in both “ara_original” and “ara_new”, but in the former it indeed differentiates between AP1 and CAL whereas in the latter both AP1 and CAL have the same motif at this position.

AG Q126H

AGAMOUS (AG) belongs to a monophyletic clade consisting of four closely related proteins in *Arabidopsis* [15]. This particular mutation was introduced because it would remove a motif (predicted by the “ara_original” model) in AG that is not present in SHP1 and SHP2, two closely related MADS proteins with somewhat different interaction partners.

A detailed description of the results from the yeast two-hybrid analyses with the above mentioned mutated proteins is given in Table S6. We also analyzed known mutations influencing MADS interactions and/or functions (Table S7). In contrast to our approach, most mutations described in literature are not aimed at changing protein-protein interaction specificity but rather at dissecting residues which are important in general for MADS functions. These need not to be related to determinants of interaction specificity; e.g. if certain residues are involved in interactions and are conserved in all MADS proteins, they are certainly not involved in interaction specificity determination. A few studies have previously applied computational approaches in order to determine residues which are under functional constraint; these might include residues which are important for protein interaction specificity (Table S8).

**3. Neo- or sub-functionalization deteriorates performance of sequence similarity-based interaction prediction but not of motif-based prediction.** As reflected by the varying interaction partners of SEP-orthologs, in different species different sub- or neo-functionalization routes have occurred [16-18]. Overall sequence similarity does not capture this, but our motifs are able to deal with this (Table S4). To obtain further insight into sub- or neo-functionalization of SEP homologs, we analyzed data from Figure 3 in [19], where various SEP equivalents are categorized according to different expression patterns in among others the outer floral whorl. Using a decision tree to select IMSS motifs that are correlated to expression in this whorl, a single motif was found; interestingly, this motif occurs at the ‘hotspot’ region discussed in the main text. Table S9 groups the SEP equivalents according to their expression in whorl 1, and indicates the sequence at the position of the motif. These results suggest that the different expression patterns are correlated with different protein-protein interaction patterns (as encoded in the sequence via the interaction motifs), thus leading to different functions.

**4. Intron borders and alternative splicing.** Analysis of motif positions relative to intron-exon borders indicates that a couple of motifs are found exactly at these borders. The placing of motifs close to exon borders might indicate a possible evolutionary mechanism to generate diversity by shifting these borders. We analyzed the distribution of distances to the closest exon border for each motif occurrence and compared it with what is obtained with random motif occurrences (Figure S1). The number of motif occurrences immediately adjacent to exon borders is slightly higher in the IMSS motifs compared to the random motif occurrences. However, the most striking aspect is the large number of motifs far away from the intron/exon border. There is a relation between the number of proteins in which a motif occurs and the distance of the motif to the exon border: motifs which occur in less than 10 MADS proteins (9 motifs) have on average a distance 2.6 (+/- 3.7) amino acids from the exon border, whereas motifs found in more than 10 proteins (11 motifs) are on average at distance 7.4 (+/- 3.1) amino acids. This suggests that motifs are more ‘stable’ when they are further away from the exon border, in line with the above proposed mechanism of generating diversity by shifting exon borders.

In case of the SVP splicing variant named SVP3 (SVP1 EFCSSS56-61D), a predicted motif at an exon border is spliced out, resulting in loss of interactions (Figure S2). An other example where splicing removes predicted interaction motifs is SEP4-I vs SEP4-II (Figure S2).

**5. Overlap between indels and interaction motifs.** To obtain insight into the dynamics of interaction motifs upon duplications, we analyzed a set of 1,459 MIKC MADS sequences from 257 species (obtained from Interpro by requiring the presence of both a MADS and K-box domain, IPR002487 and IPR002100, respectively). From these, we obtained pairs of putative in-paralogs, which we defined simply as two proteins from the same species both having their highest sequence similarity with members of the same clade in *Arabidopsis* [20].

We focused on indels, as occurrence of an insertion or deletion could be interpreted as a signature of disruption of the interaction motif. For each pair of sequences, indel positions in their sequence alignments were probed by looking for stretches of length *d* with high sequence identity, and one insertion/deletion occurring. *d* was set to 6, and the cutoff for identity was set to 5 (i.e. all positions except the indel were required to be identical). With those settings, 81 pairs of in-paralogs were found with such indel. Subsequently, the overlap between those indels and the predicted interaction motifs was assessed, and 29 pairs were found with such indel overlapping with a predicted interaction motif in 20 different species (Table S11).

Strikingly, in half of the cases listed in Table S11 (15 out of 29) there is a predicted interaction motif at the indel position both in the protein with the insertion and the protein with the deletion (but these are two different motifs). This suggests that although the occurrence of indels overlapping motifs is not distinguishable from random expectation (29 out of 81 cases do overlap a predicted interaction motif, which is in agreement with the coverage of the sequences by the “all_species” model of ~30%), in those cases where an insertion or deletion occurs in an interaction motif this often leads to a viable, but different interaction motif (15 out of 29 is ~50%). This is additional evidence for the importance of these predicted interaction motifs and suggests that they play a role in sub- and neo-functionalization. In this respect, it is important to note that although for most of these proteins no functional data is available, for some it is known that their interaction patterns are quite different between the paralogs, as is e.g. the case for the *Arabidopsis* proteins in Table S11.

Almost all of these IMSS-motif overlapping indels occur either around the ‘hotspot-motif’ (see main text), or in the C-term region. The only exception is the indel in *Hordeum vulgare* Q8RU43, *Oryza sativa* Q8RU31 and *Zea mays* Q84V75, which is at a homologous position within the K-domain in all three sequences. Note that in the *Hordeum vulgare* and *Zea* *m*aize proteins the deletion is predicted to remove an interaction motif, whereas in the *Oryza sativa* protein it is predicted to change it into another interaction motif. Importantly, this indel falls inside a motif which was experimentally validated; there is only one amino acid distance between the indel and the mutation site in AG (Q126H).

**References**

*1. de Folter S, Immink RGH, Kieffer M, Parenicova L, Henz SR, et al. (2005) Comprehensive interaction map of the Arabidopsis MADS box transcription factors. Plant Cell 17: 1424-1433.*

*2. Ciannamea S, Busscher-Lange J, de Folter S, Angenent GC, Immink RGH (2006) Characterization of the vernalization response in Lolium perenne by a cDNA microarray approach. Plant and Cell Physiology 47: 481-492.*

*3. Cseke LJ, Ravinder N, Pandey AK, Podila GK (2007) Identification of PTM5 protein interaction partners, a MADS-box gene involved in aspen tree vegetative development. Gene 391: 209-222.*

*4. Fornara F, Parenicova L, Falasca G, Pelucchi N, Masiero S, et al. (2004) Functional characterization of OsMADS18, a member of the AP1/SQUA subfamily of MADS box genes. Plant Physiology 135: 2207-2219.*

*5. Kane NA, Danyluk J, Tardif G, Ouellet F, Laliberte J-F, et al. (2005) TaVRT-2, a Member of the StMADS-11 Clade of Flowering Repressors, Is Regulated by Vernalization and Photoperiod in Wheat. Plant Physiol 138: 2354-2363.*

*6. Shan HY, Su KM, Lu WL, Kong HZ, Chen ZD, et al. (2006) Conservation and divergence of candidate class B genes in Akebia trifoliata (Lardizabalaceae). Development Genes and Evolution 216: 785-795.*

*7. Shitsukawa N, Tahira C, Kassai KI, Hirabayashi C, Shimizu T, et al. (2007) Genetic and epigenetic alteration among three homoeologous genes of a class E MADS box gene in hexaploid wheat. Plant Cell 19: 1723-1737.*

*8. Sundstrom J, Engstrom P (2002) Conifer reproductive development involves B-type MADS-box genes with distinct and different activities in male organ primordia. Plant Journal 31: 161-169.*

*9. Parenicova L, de Folter S, Kieffer M, Horner DS, Favalli C, et al. (2003) Molecular and phylogenetic analyses of the complete MADS-box transcription factor family in Arabidopsis: New openings to the MADS world. Plant Cell 15: 1538-1551.*

*10. Yant L, Mathieu J, Schmid M (2009) Just say no: floral repressors help Arabidopsis bide the time. Current Opinion in Plant Biology 12: 580-586.*

*11. Hartmann U, Hohmann S, Nettesheim K, Wisman E, Saedler H, et al. (2000) Molecular cloning of SVP: a negative regulator of the floral transition in Arabidopsis. Plant journal 21: 351-360.*

*12. Li D, Liu C, Shen L, Wu Y, Chen H, et al. (2008) A repressor complex governs the integration of flowering signals in Arabidopsis. Developmental Cell 15: 110-120.*

*13. Kempin SA, Savidge B, Yanofsky MF (1995) Molecular-Basis of the Cauliflower Phenotype in Arabidopsis. Science 267: 522-525.*

*14. Alvarez-Buylla ER, Garcia-Ponce B, Garay-Arroyo A (2006) Unique and redundant functional domains of APETALA1 and CAULIFLOWER, two recently duplicated Arabidopsis thaliana floral MADS-box genes. Journal of Experimental Botany 57: 3099-3107.*

*15. Favaro R, Pinyopich A, Battaglia R, Kooiker M, Borghi L, et al. (2003) MADS-box protein complexes control carpel and ovule development in Arabidopsis. Plant Cell 15: 2603-2611.*

*16. Ferrario S, Immink RGH, Shchennikova A, Busscher-Lange J, Angenent GC (2003) The MADS box gene FBP2 is required for SEPALLATA function in petunia. Plant Cell 15: 914-925.*

*17. Uimari A, Kotilainen M, Elomaa P, Yu DY, Albert VA, et al. (2004) Integration of reproductive meristem fates by a SEPALLATA-like MADS-box gene. Proceedings of the National Academy of Sciences of the United States of America 101: 15817-15822.*

*18. Ruokolainen S, Ng YP, Albert V, Elomaa P, Teeri T (2010) Large scale interaction analysis predicts that the Gerbera hybrida floral E function is provided both by general and specialized proteins. BMC Plant Biology 10: 129.*

*19. Malcomber ST, Kellogg EA (2005) SEPALLATA gene diversification: brave new whorls. Trends in Plant Science 10: 427-435.*

*20. Becker A, Theissen G (2003) The major clades of MADS-box genes and their role in the development and evolution of flowering plants. Molecular Phylogenetics and Evolution 29: 464-489.*
